# Cytotoxicity induced by *Aeromonas schubertii* is orchestrated by a unique set of type III secretion system effectors

**DOI:** 10.1186/s13567-025-01548-2

**Published:** 2025-06-08

**Authors:** Hana Michova, Jan Pliva, Anezka Jirsova, David Jurnecka, Jana Kamanova

**Affiliations:** 1 Laboratory of Infection Biology, Institute of Microbiology, Czech Academy of Sciences, Videnska 1083, Prague, 142 00 Czech Republic; 2 Laboratory of Molecular Biology of Bacterial Pathogens, Institute of Microbiology, Czech Academy of Sciences, Videnska 1083, Prague, 142 00 Czech Republic

**Keywords:** *Aeromonas*, *Aeromonas schubertii*, type III secretion system effectors, cytotoxicity, VopQ, ExoY

## Abstract

The type III secretion system (T3SS) is an important virulence factor of Gram-negative bacteria, including the genus *Aeromonas*, which represents a diverse group of aquatic bacteria. One member of the genus, *Aeromonas schubertii*, is an emerging pathogen in aquaculture, causing high mortality in snakehead fish. Infections are associated with the formation of white nodules in the internal organs, likely resulting from *A. schubertii*-induced apoptosis and/or necrosis. The present study investigates the type strain *A. schubertii* ATCC 43700, which encodes two distinct T3SSs located within *Aeromonas* pathogenicity islands 1 and 2, referred here to as API1 and API2. We analyzed their role in *A. schubertii*-induced cytotoxicity and identified novel T3SS effector proteins. Infections of HeLa cells revealed that API1, but not API2, mediates cytotoxicity and induces both apoptotic and necrotic cell death. Moreover, proteomic analysis identified seven candidate effectors secreted by the API1 injectisome. These included two previously described effectors, AopH and AopO from *A.* *salmonicida*, as well as five novel effectors named AopI, AopJ, AopL, AopT, and AopU, whose injection into host cells was validated using a split luciferase reporter system. Functional characterization showed that AopL, a homolog of *Vibrio parahaemolyticus* VopQ, induces caspase-3/-7-independent necrosis, while AopI, a homolog of ExoY from *Pseudomonas aeruginosa*, suppresses caspase-3/-7 activation and necrosis, revealing a pro-survival function. These results demonstrate the critical role of the API1 injectisome in *A. schubertii*-induced cytotoxicity and provide experimental identification of novel *Aeromonas* effectors that cooperate to fine-tune host cell cytotoxicity.

**Supplementary Information:**

The online version contains supplementary material available at 10.1186/s13567-025-01548-2.

## Introduction

Members of the genus *Aeromonas* are ubiquitous bacteria that are generally associated with aquatic environments and form long-term mutualistic relationships in the gastrointestinal tract of fish and leeches [1–3]. However, they are also important fish pathogens that cause significant economic losses in aquaculture [4]. In addition, *Aeromonas* species are increasingly recognized as opportunistic pathogens in humans, causing a plethora of symptoms ranging from mild gastroenteritis to severe necrotic fasciitis or septicemia in immunocompromised individuals [5–9]. The genus is genetically diverse and highly adaptable, due in part to its exceptional ability to acquire mobile genetic elements such as plasmids, transposons, and pathogenicity islands. It is often described as a genetic “sponge” that is able to take up and spread antibiotic resistance and virulence genes through horizontal gene transfer [10–12]. Virulence factors include structural components such as flagella, pili, and capsules, as well as a variety of secreted toxins and enzymes that impair host defense. Among these, the type III secretion system (T3SS) and its effector proteins play a central role in the virulence of several *Aeromonas* species [13, 14].

The T3SS is a sophisticated nanomachine, often referred to as the T3SS injectisome, that translocates effector proteins directly into host cells where they manipulate host processes to the advantage of the bacteria. Structurally, the injectisome consists of a basal body, a needle-like protrusion, and a translocon that forms a pore in the membrane of the host cell. The genes encoding these nanomachines are categorized into seven families and are located on mobile pathogenicity islands that facilitate horizontal gene transfer [15, 16]. A bacterium can carry more than one T3SS island. *Salmonella enterica*, for example, encodes two distinct T3SS pathogenicity islands, called SPI1 and SPI2, which have different functions. While SPI1 is required for bacterial invasion into non-phagocytic cells, SPI2 is activated intracellularly and interferes with phagosomal maturation [17]. In contrast, the genes encoding effector proteins are more diverse and can be acquired independently, leading to their scattered distribution across the genome.

Previous studies have mainly focused on the Ysc family of T3SS injectisomes and their effector proteins in non-motile *A. salmonicida* as well as in the motile mesophilic species *A. hydrophila*, *A. piscicola*, and *A.* *dhakensis* [16, 18]. In *A. salmonicida*, the T3SS injectisome is encoded on a plasmid, and its presence is essential for the virulence of *A. salmonicida* in cold-water fish, with mutants exhibiting lower virulence [19, 20]. The injected effector proteins have diverse biochemical activities, and their interactions influence cell viability, host immune responses, and cytoskeletal dynamics. They include the bifunctional ADP-ribosylation GTPase-activating effector AexT [21, 22], the phosphatidylinositol phosphatase Ati2 [23], the putative acetyltransferase AopP [24], and the putative protein tyrosine phosphatase AopH and serine/threonine kinase AopO [25, 26]. In *A. hydrophila*, *A. dhakensis* (formerly *A. hydrophila* SSU) and *A. piscicola* (formerly A*. hydrophila* AH-3), T3SS-positive strains also show increased virulence, while mutants exhibit reduced cytotoxicity and increased susceptibility to phagocytosis [27–30]. However, only two effectors have been identified so far. The actin filament-disrupting AexT, which is homologous to AexT from *A. salmonicida*, and the functionally similar AexU [31–34]. Interestingly, a recent bioinformatic analysis of T3SS loci and effector proteins in the genomes of 105 *Aeromonas* strains revealed high variability and identified numerous potential effectors that showed varying levels of cytotoxicity when expressed in the yeast *Saccharomyces cerevisiae* [35]. The analysis also revealed that some *Aeromonas* strains possess two distinct T3SS loci [35], hereby referred to as *Aeromonas* pathogenicity island 1 (API1) and 2 (API2), whose specific functions remain to be established.

The presented work focuses on *A. schubertii*, which mainly infects aquatic animals. Infections with high mortality rates have been reported in commercially important species, such as whiteleg shrimp [36], tilapia [37], and Asian seabass [38]. In addition, since 2009, multidrug-resistant *A. schubertii* strains have caused devastating infections in snakehead fish, *Channa maculata*, *C. argus*, and their hybrids, which are farmed extensively in southern China [39–41]. The infection, known as “internal white spot disease”, causes the formation of white nodules in the spleen, liver, and kidneys. The nodules resemble mycobacterial granulomas and are likely the result of *A. schuberti*-induced apoptosis and/or necrosis [42, 43]. In addition to its impact on aquatic species, *A. schubertii* is also an opportunistic pathogen for humans, associated with abscesses, wound infections, gastroenteritis, and sepsis [44, 45]. Intriguingly, *A. schubertii* lacks most of the T3SS effectors identified in other *Aeromonas* species, and the mechanisms responsible for its pathogenicity are poorly understood. Therefore, the aim of this study was to characterize the role of API1- and API2-encoded injectisomes in *A. schubertii* infection using a HeLa cell model and to identify effector proteins injected into host cells.

## Materials and methods

### Bacterial strains and growth conditions

The type strain of *Aeromonas schubertii* ATCC 43700 (also known as CECT4240 or CDC 2446-81), originally isolated from a human forehead abscess in Texas [44, 46], together with its derived mutant strains, were used throughout this study. A detailed list of strains is provided in Additional file 1. Bacteria were cultivated on tryptone soya agar (TSA; Oxoid) at 30 °C for 48 h following inoculation from stocks preserved in 40% glycerol at −80 °C. Liquid cultures of *A. schubertii* strains were grown in tryptone soya broth (TSB; Oxoid) at 30 °C with constant shaking at 180 rpm. For experiments, overnight cultures in TSB were subcultured at a 1:25 ratio into fresh TSB and incubated for approximately 4 h to reach the exponential growth phase unless stated otherwise. For plasmid construction, *Escherichia coli* strain XL-1 Blue was used, while *E. coli* strain SM10λ pir was employed for plasmid transfer into *A. schubertii* via bacterial conjugation. *E. coli* strains carrying the temperature-sensitive allelic exchange vector pAX2 were cultured at 30 °C on LB agar or in LB broth. When appropriate, LB media were supplemented with 100 µg/mL ampicillin.

### Plasmid construction and generation of *A. schubertii* mutant strains

Mutant strains of *A. schubertii* ATCC 43700, including in-frame deletion mutants and HiBiT-tagged strains, were generated through homologous recombination using the pAX2 allelic exchange vector (gift from Karen Guillemin; Addgene plasmid # 117398; [47]). Details of pAX2-derived plasmids used in this study are listed in Additional file 2. Plasmids were constructed using the Gibson assembly method [48]. First, two homologous regions, H1 and H2, of chromosomal DNA of *A. schubertii* ATCC 43700 with overlaps homologous to pAX2 vector were amplified with Herculase II Phusion DNA polymerase (Agilent) using primers listed in Additional file 3 (annealing temperature 58 °C). Fragments were purified by agarose gel electrophoresis and were subsequently ligated into the *Sma*I-linearized pAX2 vector. Constructed plasmids were verified by DNA sequencing (Eurofins Genomics) before being introduced into *A. schubertii* by bacterial conjugation. For conjugation, *A. schubertii* and *E. coli* SM10λ pir strain were mixed in a 1:1 ratio on a filter disk and placed on TSA. The mating mixture was incubated overnight at 30 °C. After the incubation, bacteria were recovered and plated on TSA supplemented by gentamicin (10 µg/mL) and anhydrotetracycline (10 ng/mL) to select for *A.* *schubertii* merodiploids, as described previously [47]. Isolated merodiploid colonies were screened for the loss of GFP expression indicating the second recombination event. PCR genotyping was performed to differentiate between wild-type and mutant alleles.

### Mammalian cell culture

Cell lines HeLa (ATCC CCL-2, human cervical adenocarcinoma) and HeLa-LgBit (HeLa cells constitutively expressing LgBit; [49]) were cultivated in Dulbecco’s Modified Eagle Medium supplemented with 10% (vol/vol) heat-inactivated fetal bovine serum (DMEM-10% FBS) at 37 °C and 5% CO_2_.

### Time-lapse HeLa live cell imaging

To visualize host cells during *A. schubertii* infection, 1 × 10^5^ of HeLa cells in DMEM-10% FBS were seeded in each well of a 4-well glass-bottom dish (Cellvis) and allowed to adhere overnight. The next day, cells were either left uninfected, infected with *A. schubertii* wild type (WT) or its ΔAPI1 and ΔAPI2 derivatives (see Additional file 1) at MOI of 10:1. To enhance infection efficiency, the dish was briefly centrifugated (5 min; 120 *g*). One hour post-infection, gentamicin was added to a final concentration of 100 µg/mL to stop the infection, and the dish was transferred to a prewarmed stage-top incubation chamber of a motorized fluorescence microscope (IX-83, Olympus, Japan) equipped with a sCMOS camera Photometrics Prime 95B. Bright-field images were acquired using a 40 × dry objective (UPLXAPO40X, NA = 0.95) under controlled conditions of 37 °C and 5% CO_2_. Sequential 16-bit images were acquired as a time-lapse of 24 h with frame intervals of 10 min using the CellSens software. Images were then processed with ImageJ (Fiji, [50]) by enhancing the brightness to contrast ratio, selecting ROI, and adding a scale bar.

### Determination of caspase 3 and/ or caspase 7 activation using Caspase-Glo assay

To evaluate caspase-3 and/or -7 activation during infection, HeLa cells (1.5 × 10^4^ per well) were seeded in a 96-well plate in DMEM-10% FBS. The next day, *A. schubertii* strains grown to exponential phase were washed twice with DMEM-10% FBS by centrifugation (3 min; 8000 *g*) and added to cells at MOI 10:1. The plate was centrifuged (5 min; 120 *g*) to enhance the infection efficiency. One hour post-infection, infection was stopped by the addition of gentamicin (100 µg/mL), and cells were further incubated at 37 °C with 5% CO_2_. At designated time points, an equal volume of freshly reconstituted Caspase-Glo 3/7 Reagent (Promega, Cat. No. G8091), containing a pro-luminescent caspase-3/7 substrate, was added to each well. The plate was then incubated for 30 min at 37 °C to allow cell lysis and substrate cleavage by activated caspases, generating a luminescent signal. Following the incubation, 100 µL of the reaction mixture from each well was transferred to a 96-well white/clear-bottom plate (Corning). Luminescence was measured using the Spark microplate reader (Tecan) with an integration time of 1 s per well. Experimental conditions included both uninfected cells and a positive control treated with 1 µM staurosporine. For statistical analysis, data were normalized by the average luminescence of WT-infected samples and compared using two-tailed unpaired parametric *t*-test.

### Analysis of caspase 3 activation by western blot

HeLa cell were seeded in 6-well cell culture plate (5 × 10^5^ per well), and infected the following day with *A. schubertii* strains washed in DMEM-10%FBS at MOI 10:1. The plate was centrifugated (5 min; 120 *g*), and infection was terminated after 1 h by addition of gentamicin (100 µg/mL). At indicated time points, the content of each well was aspirated, and cells were then lysed by addition of 100 µL of lysis buffer consisting of 0.3% (vol/vol) Triton X-100 in PBS containing the Complete Protease Inhibitor Cocktail (Roche). Extracted proteins were boiled (95 °C; 5 min), separated by 15% SDS-PAGE electrophoresis, and transferred onto a nitrocellulose membrane. Membranes were probed overnight with rabbit polyclonal antibody against caspase-3 (dilution 1:1000; CST, Cat. No. 9662). The detected caspase-3 was revealed with 1:3000-diluted horseradish peroxidase (HRP)-conjugated anti-rabbit IgG secondary antibodies (GE Healthcare) using a Pierce ECL chemiluminescence substrate (Thermo Fisher Scientific) and Image Quant LAS 4000 station (GE Healthcare). For loading control, membranes were re-probed with mouse monoclonal IgG against β-actin (dilution 1:10 000; Proteintech, Cat. No. 66009-1-Ig) and revealed with secondary HRP-anti-mouse IgG (Cytiva), as above.

### Determination of plasma membrane permeabilization using CellTox Green assay

HeLa cells (2 × 10^4^ per well) were seeded in a 96-well black/clear-bottom plate (Corning) in DMEM-10% FBS. The next day, *A. schubertii* and the derived mutant strains were washed in DMEM-10% FBS and added to the wells at the indicated MOI. The plate was then centrifugated (5 min; 120*g*) to facilitate bacterial contact with cells. One hour post-infection, gentamicin (100 µg/mL) and the fluorescent DNA-binding dye CellTox Green (Cat. No. G8743, Promega) were added to each well. The plate was subsequently incubated under controlled conditions (37 °C and 5% CO_2_) inside the Spark microplate reader (Tecan). Fluorescence measurements were recorded at 15 min intervals over a 24 h using excitation and emission wavelengths of 490 nm and 525 nm, respectively. For statistical analysis, the time at which the WT signal reached half of its maximum was compared to the corresponding time for mutant strains using a two-tailed unpaired parametric *t*-test.

### Proteomic analysis of secretomes

Bacterial cultures were grown overnight in 50 mL of TSB medium with or without supplementation of 0.5 mM EGTA and 20 mM MgCl_2_ at 30 °C with constant shaking of 180 rpm. Subsequently, cells were removed by centrifugation (30 min; 10 000 *g*; 4 °C) followed by filtration through 0.22 μm membranes. The resulting supernatants containing secreted proteins were precipitated by adding trichloracetic acid (Sigma) to a final concentration of 10% (v/v) and incubating at 4 °C overnight. Precipitated proteins were collected by centrifugation (30 min; 14 000 *g*; 4 °C), washed twice with cold acetone, air-dried, and dissolved in 50 mM ammonium bicarbonate with 8 M urea. Protein concentrations were determined using the Pierce BCA Protein Assay Kit (Thermo Fisher Scientific), and 70 µg of protein per sample was used for further processing. Samples were analyzed by label-free mass spectrometry (MS) using a nanoLC-MS/MS system coupled to an Orbitrap Fusion Tribrid mass spectrometer (Thermo Fisher Scientific) as previously described [51]. Data were analyzed and quantified using a label-free quantification approach with MaxQuant (version 2.5.2.0) and Perseus (version 2.0.11). The false discovery rate (FDR) was set to 1% for both proteins and peptides. The enzyme specificity of trypsin was set as C-terminal to Arg and Lys residues. Carbamidomethylation was set as a fixed modification, while N-terminal protein acetylation and methionine oxidation were considered variable modifications. The maximum number of missed cleavages was set to two. Protein identification was performed using the *Aeromonas schubertii* reference proteome database (*Aeromonas schubertii* ATCC 43700, Uniprot—UP000054876). Statistical analysis was conducted in Perseus using a Student *t*-test with a significance threshold of *p* < 0.01, and only proteins with a fold change ≥ 4 were considered upregulated. The mass spectrometry proteomics data have been deposited to the ProteomeXchange Consortium via the PRIDE [52] partner repository with the dataset identifier PXD062075.

### Quantification of intracellular and secreted levels of candidate effectors by luminescence measurements

To evaluate the levels of secreted and intracellular effectors, reporter strains expressing HiBit-tagged candidate effectors and their ΔAPI1 and ΔAPI2 derivatives (see Additional file 1) were cultivated in TSB medium with or without supplementation of 0.5 mM EGTA and 20 mM MgCl_2_ to reach OD_600nm_ 1 ± 0.05. One mL of each culture was centrifuged (5 min; 8000 *g*) and levels of candidate effectors were quantified in both the supernatants representing the secreted fraction, and in the cell pellet extracts containing intracellular effectors. The quantification was performed using the Nano-Glo HiBiT Extracellular Detection System (Promega, Cat. No. N2420) as previously described [49].

In brief, for the preparation of cell extracts, cell pellets were resuspended in 200 µL of extraction buffer (150 mM NaCl in 50 mM Tris–HCl, pH 8.0) and combined with 0.1 mm glass beads (Scientific Industries). Cells were lysed using the Disruptor Genie (Scientific Industries) with two cycles of bead beating (3 min at maximum speed), followed by a cooling interval of 3 min on ice. The disrupted cell suspension was diluted with 800 µL of the same buffer and clarified by centrifugation (10 min; 14 000 *g*). Cell culture supernatants and pellet extracts were subsequently transferred into 96-well white/clear-bottom plate and mixed with an equal amount of reconstituted Nano-Glo HiBiT Extracellular Reagent containing recombinant LgBit protein and furimazine substrate in Nano-Glo buffer. Luminescence was measured using the Spark microplate reader (Tecan) with an integration time of 1 s per well. To determine the exact concentration of effectors, serial dilutions of purified recombinant LRT-HiBiT protein were included in each plate as a standard [49].

### Determination of effector injection into the host cell

To evaluate the injection of candidate effectors into the host cells, 2 × 10^4^ of HeLa-LgBit (HeLa cells constitutively expressing LgBit) per well were seeded in a 96-well white/clear-bottom plate (Corning), in DMEM-10% FBS. The next day, reporter strains expressing HiBit-tagged candidate effectors and their ΔAPI1 and ΔAPI2 derivatives (see Additional file 1) grown to exponential phase were washed twice in DMEM-10%FBS by centrifugation (5 min; 8000 *g*) and added to cells at MOI of 50:1, along with Nano-Glo Live Cell Reagent (Promega, Cat. No. N2011) containing cell permeable luciferase substrate in Nano-Glo buffer. After centrifugation (5 min; 120 *g*), the plate was placed inside the chamber of the Spark microplate reader (Tecan) with 37 °C and 5% CO_2_, and luminescence measurements were performed for 2 h at 3 min intervals with an integration time of 1 s. The maxima of the signal reached for each tagged effector were normalized as a fold change towards the uninfected control and were statistically evaluated using two-tailed unpaired parametric *t*-test.

### Bioinformatic analysis

The genome sequence of *A. schubertii* ATCC 43700 was downloaded from NCBI (BioProject PRJNA304368, NCBI RefSeq: GCF_001481395.1, [46]). Homology of T3SS-related proteins and potential effectors was established using Protein BLAST run against non-redundant protein sequences of *Aeromonas salmonicida*, *Aeromonas veronii*, *Yersinia* spp., *Vibrio parahaemolyticus*, *Pseudomonas aeruginosa*, *Salmonella enterica*, and *Bordetella* spp. Only proteins with the highest Percent Identity were taken into consideration.

To create a phylogenetic tree, sequences of SctN and SctC from various T3SS-expressing bacteria were downloaded from the Uniprot database (see Additional file 4). Sequences were aligned, and their phylogenetic tree was constructed as a Minimum Evolution Tree based on p-distance using MEGA 11 software [53]. Trees were further annotated using the online tool iTOL v7 [54].

To create sequence alignment, amino acid sequences were aligned using the Clustal Omega online alignment tool available at the Uniprot website and visualized with the percentage identity scheme in Jalview [55].

## Results

### *A. schubertii* carries two distinct T3SS injectisomes belonging to the Ysc and Ssa-Esc families

**Figure 1:**
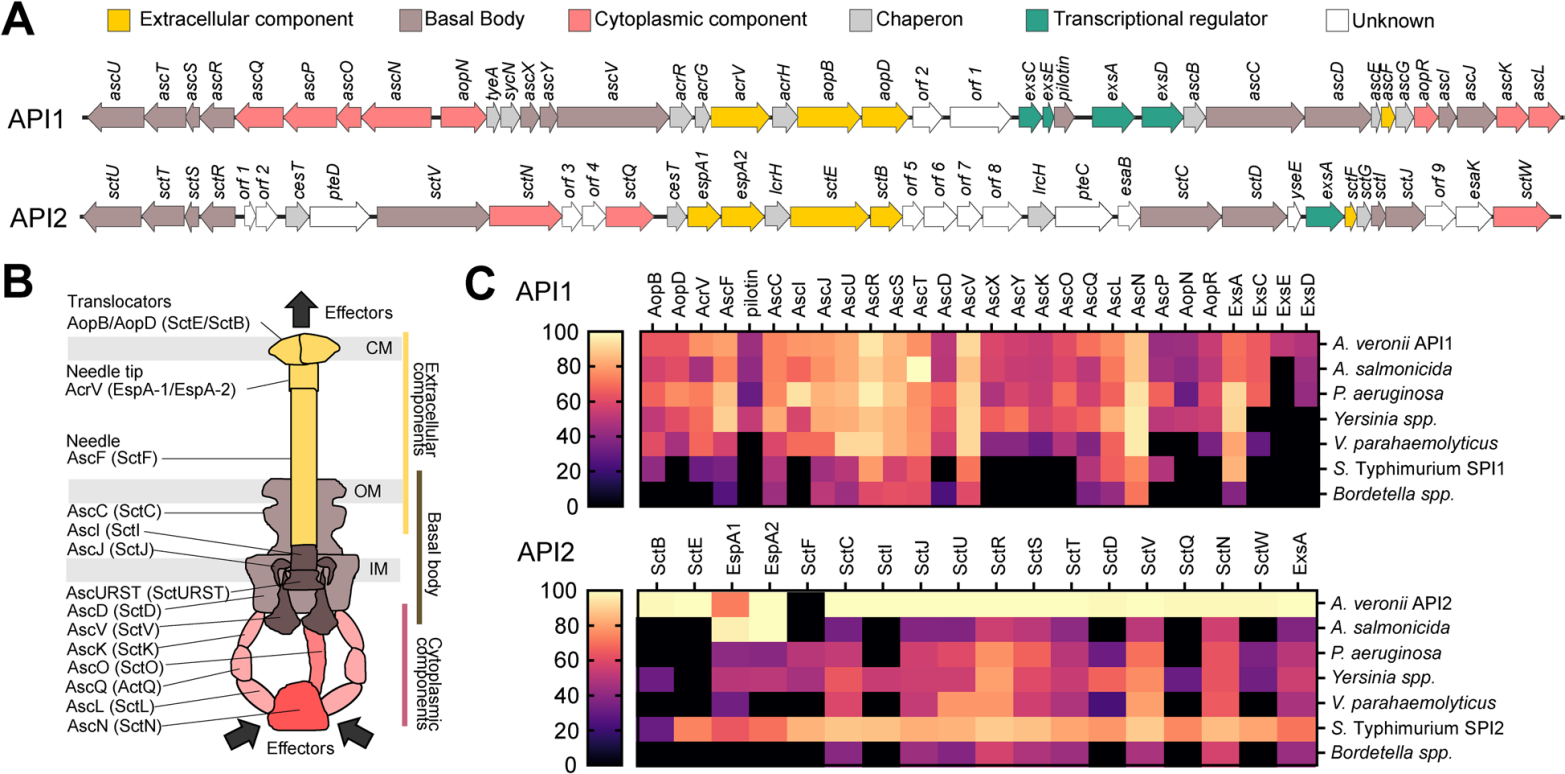
***Aeromonas schubertii *ATCC 43700 harbors two distinct loci encoding T3SS injectisomes.** **A** Genetic organization of the API1 and API2 loci in *A. schubertii*. **B** Predicted structural organization of the T3SS injectisomes encoded by API1 and API2 loci. The diagram illustrates predicted localization of the proteins corresponding to the genes shown in panel **A**. **C** Similarity of proteins encoded in the API1 and API2 loci to T3SS components from the indicated bacterial species. The heatmap displays the percentage similarity of each protein to its corresponding T3SS ortholog.

The type strain *Aeromonas schubertii* ATCC 43700 (also known as CECT4240 or CDC 2446–81) harbors two different T3SS loci located within *Aeromonas* pathogenicity islands 1 (API1) and 2 (API2), which encode two distinct injectisomes (Figure 1A). Each locus contains all the essential structural proteins required for the formation of a functional T3SS injectisome (Figure 1B), although they exhibit differences in their genetic architecture and the proteins they encode. Importantly, the majority of proteins encoded by API1 show a high degree of similarity to well-characterized T3SS proteins from different bacterial species, whereas API2 contains a variety of hypothetical proteins with poorly characterized properties. In addition, similarity assessments and phylogenetic analyses, shown in Figure 1C and in Additional file 5, indicate that API1 belongs to the Ysc injectisome family, which is widely distributed within the genus *Aeromonas* and shows high similarity to the injectisomes of *Yersinia* spp. and *Pseudomonas aeruginosa*. In contrast, API2 shows homology with the injectisome of *Salmonella enterica*, which belongs to the Ssa-Esc family and is encoded on the *Salmonella* pathogenicity island 2 (SPI2).

### API1 injectisome is required for cellular cytotoxicity of *A. schubertii*

**Figure 2:**
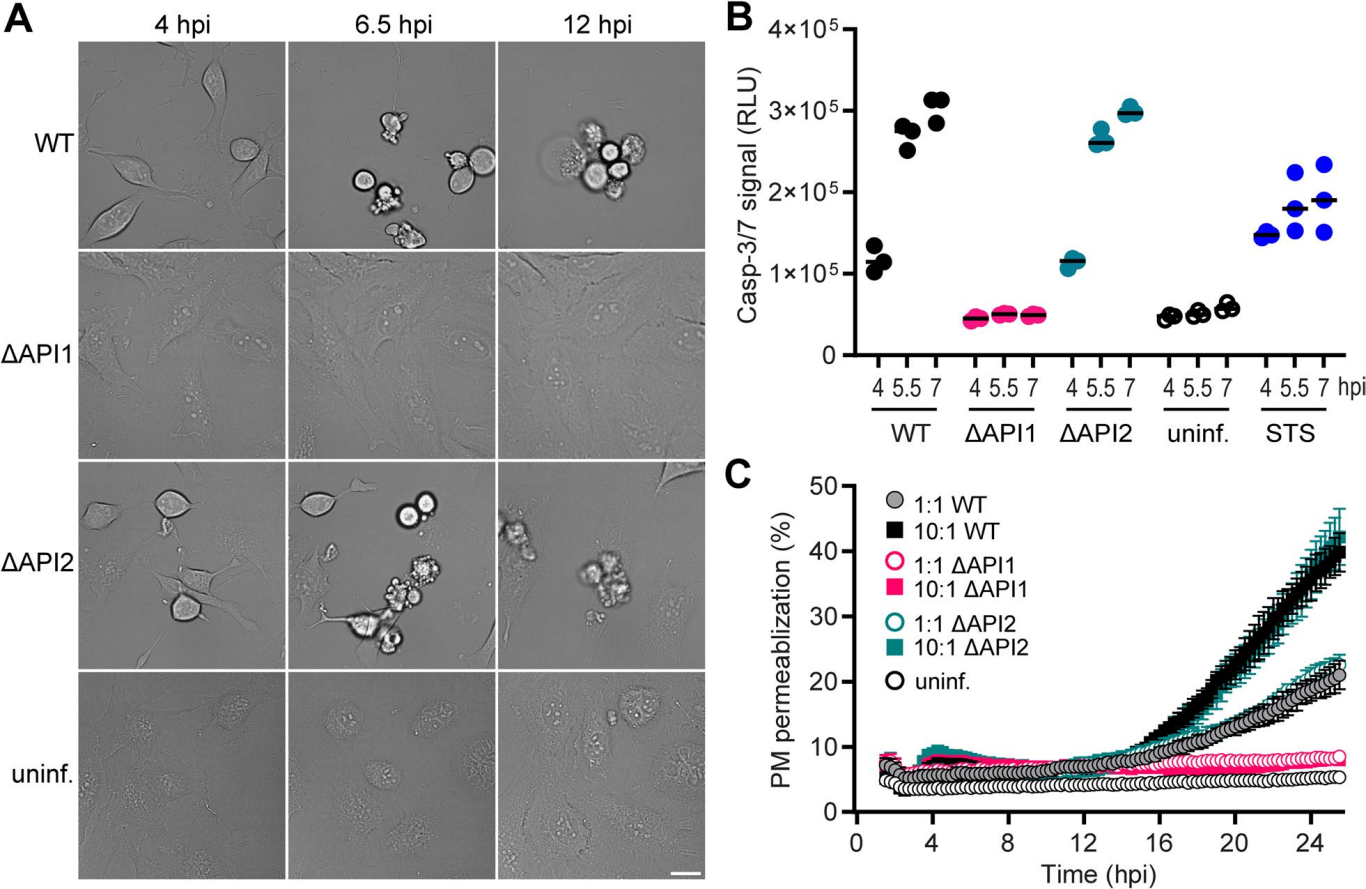
**API1 injectisome is required for cellular cytotoxicity of *A. schubertii*.** HeLa cells were either left uninfected or infected with *A. schubertii* wild-type (WT) or mutant strains lacking the API1 (ΔAPI1) or API2 (ΔAPI2) injectisomes, due to the deletion of the respective T3SS ATPases, at MOI 10:1. One hour post-infection, the extracellular bacteria were eliminated by the addition of gentamicin. **A** HeLa cells were analyzed by live-cell imaging. A sequence of time-lapse images is shown. Data are representative of 3 independent experiments. Scale bar, 20 µm. **B** Activation of caspase-3 and/or caspase-7 in infected HeLa cells was determined at indicated time points using the Caspase-Glo 3/7 assay, which detects cleavage of a proluminescent caspase-3/7 substrate. Data are presented as individual luminescence values (dots) from a representative experiment performed in triplicate wells out of 2 independent experiments. The black bar represents the mean. HeLa cells treated with 1 µM staurosporine (STS) served as a positive control. **C** Real-time kinetics of plasma membrane (PM) permeabilization in infected HeLa cells was assessed using the fluorescent DNA-binding dye CellTox Green. PM permeabilization is expressed relative to complete permeabilization induced by a cell lysis solution. Data represent the mean ± SD of triplicate wells and are representative of 2 independent experiments.

To clarify the functions of the injectisomes API1 and API2 in the pathogenesis of *A. schubertii*, in-frame deletions for the ATPase genes *ascN* (API1) and *sctN* (API2) were generated, resulting in the mutant strains designated ΔAPI1 and ΔAPI2. These mutant strains were used together with the wild-type strain (WT) to infect HeLa cells at a multiplicity of infection (MOI) of 10:1. After 1-h infection, extracellular bacteria were eliminated by gentamicin, and time-lapse microscopy was performed. The morphological analysis revealed significant cellular changes and cytotoxicity upon infection with the WT strain, as shown in Figure 2A and Additional file 6. Early changes included rounding of cells, indicating reorganization of the cytoskeleton and/or disruption of cellular adhesion, which was followed by the appearance of apoptotic features. These events later culminated in cellular disintegration. Infection with the ΔAPI2 mutant resulted in effects similar to those of the WT strain, while HeLa cells infected with the ΔAPI1 mutant exhibited negligible morphological changes and were very similar to uninfected controls (Figure 2A and Additional file 6).

To corroborate these results, we assessed the induction of apoptosis by quantifying caspase-3/7 activity using the Caspase-Glo 3/7 assay, which measures the luminescence produced by the cleavage of a proluminescent substrate by active caspases. In addition, necrosis was assessed by analyzing the permeabilization of the plasma membrane. As shown in Figure 2B, infection with the WT and ΔAPI2 mutant strains elicited detectable caspase activation at MOI of 10:1 as early as 4 h post-infection, which is the earliest time point examined. Furthermore, the luminescence signal more than doubled within the subsequent 2-h interval, indicating increased caspase activity (Figure 2B). In contrast, cells infected with the ΔAPI1 strain showed no caspase activity, as was also observed in uninfected controls. Western blot analysis confirmed these results and showed that caspase-3 processing occurred in HeLa cells infected with the WT and ΔAPI2 strains, whereas such processing was absent in cells infected with the ΔAPI1 strain (see Additional file 7). In addition, plasma membrane permeabilization was detected in cells infected with the WT and ΔAPI2 strains at MOI of 10:1, as assessed by the fluorescent DNA-binding dye CellTox Green, but only at 16 h post-infection (Figure 2C). Membrane permeabilization was also observed at a lower MOI of 1:1, but with reduced efficiency. Both the WT and ΔAPI2 strains exhibited comparable levels of necrosis, while cells infected with the ΔAPI1 mutant showed no signs of membrane permeabilization.

Overall, these results demonstrate that the API1 injectisome is critical for the translocation of effector proteins that mediate *A. schuberti*-induced cytotoxicity. This cytotoxicity includes activation of apoptotic caspases and cell necrosis, which might be secondary necrosis following apoptosis, or necrosis independent of apoptosis. In contrast, the API2 injectisome does not contribute to cellular cytotoxicity in the HeLa model system.

### Identification of candidate effectors of the API1 injectisome

**Figure 3:**
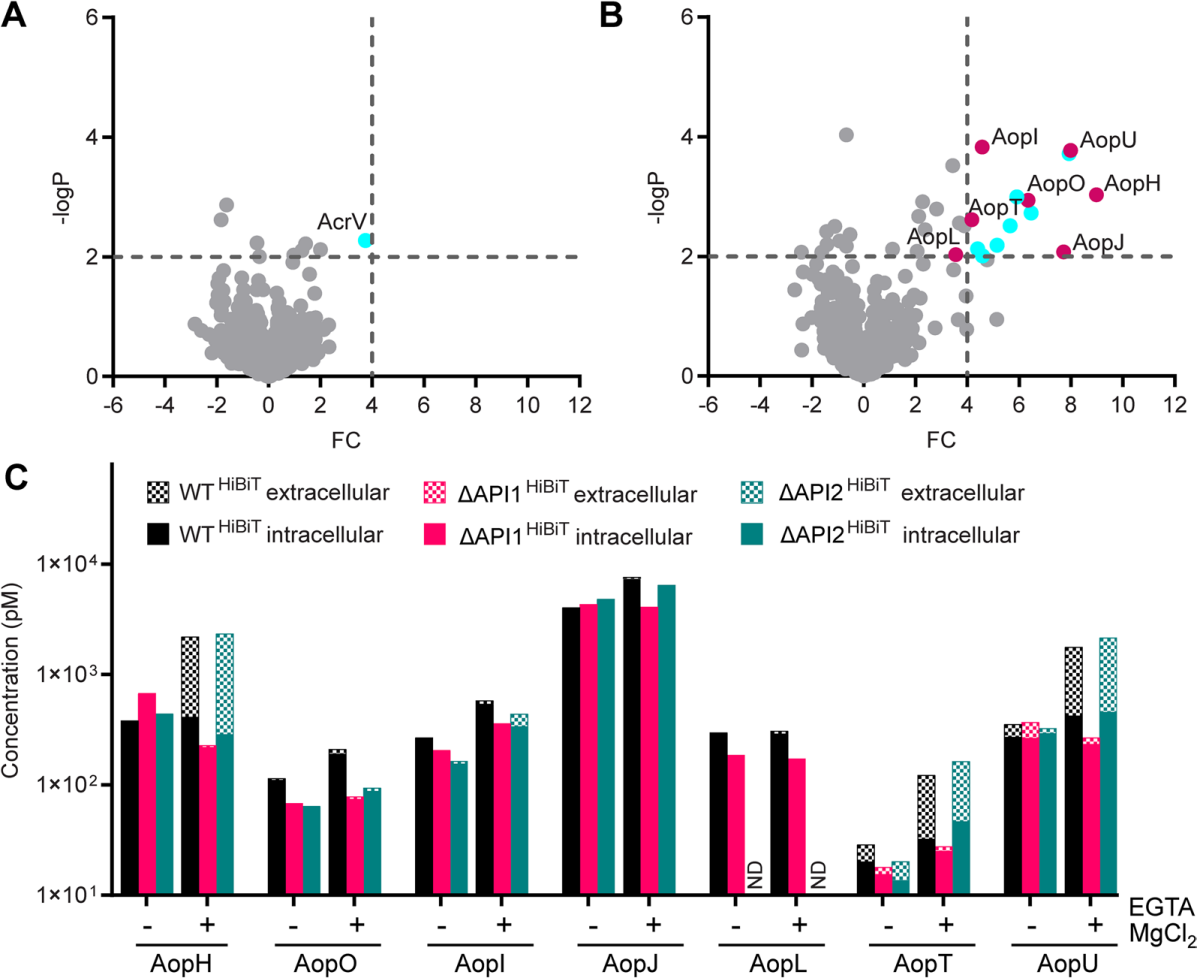
**Identification of candidate effectors of the API1 injectisome.** **A**–**B** Volcano plots illustrating global proteomic changes in the secretomes of *A.* *schubertii* wild-type (WT) compared to the ΔAPI1 mutant strain lacking the API1 injectisome. Strains were grown in TSB medium without (**A**) and with supplementation (**B**) of 0.5 mM EGTA and 20 mM MgCl_2_. Red dots indicate candidate effector proteins, while blue dots represent proteins predicted to be structural components of the injectisome. Significant changes were defined as fold change ≥ 4 and −log_10_*P* value ≥ 2, corresponding to *P* ≤ 0.01. **C** Secretion of candidate effector proteins in response to low Ca^2+^/high Mg^2+^ concentrations. Luminescence measurements were used to quantify the extracellular and intracellular levels of candidate effector proteins. Measurements were performed on WT reporter (WT^HiBiT^) strains and the corresponding mutant derivatives lacking API1 (ΔAPI1^HiBiT^) and API2 (ΔAPI2^HiBiT^) injectisomes. Cultures were grown in TSB medium without and with supplementation of 0.5 mM EGTA and 20 mM MgCl_2._ Data represent the absolute concentration of candidate effector^HiBiT^ in extracellular and intracellular fractions and are representative of 3 independent experiments. ND, not determined.

To date, no T3SS effectors have been experimentally identified in *A. schubertii*. However, bioinformatic analysis of the *A. schubertii* genome and heterologous expression studies in *S. cerevisiae* have revealed homologs of two known effectors from *A. salmonicida*, namely AopH and AopO, as well as several additional putative effectors [35]. To determine the effectors delivered by API1 that are responsible for cellular cytotoxicity, the secretomes of wild-type (WT) and ΔAPI1 strains cultivated overnight in TSB medium were subjected to mass spectrometry analysis. However, no proteins were significantly enriched in the secretome of the WT strain at the predefined thresholds, indicating that the API1 injectisome remains inactive under standard cultivation conditions (Figure 3A). This observation is consistent with the known activation mechanism of T3SS injectisomes, which are typically triggered upon contact with host cell membranes during infection. To overcome this limitation, the TSB medium was supplemented with EGTA to chelate calcium ions and MgCl_2_ to suppress the PhoPQ system, which has previously been shown to artificially activate the T3SS injectisomes [56, 57]. Under these modified conditions, the activity of the API1 injectisome was significantly increased (Figure 3B and Additional file 8). Comparative proteomic analysis identified numerous proteins at the predefined threshold that were secreted at significantly higher levels by the WT strain compared to the ΔAPI1 mutant. These included structural components of the API1 injectisome, homologs of the *A. salmonicida* effectors, AopH and AopO, as well as several candidate effectors, including previously predicted putative effectors: PteI, hereafter referred to as AopI for *Aeromonas* outer protein I, which is homologous to the nucleotidyl cyclase ExoY from *Pseudomonas aeruginosa*, and PteJ, hereafter referred to as AopJ, which is homologous to the phosphothreonine lyase OspF from *Shigella flexneri* [35] (for sequence alignment, see Additional file 9). In addition, two new candidate effectors were identified and named: AopT, characterized by the presence of a lipase domain, and AopU, which possesses a RhoGAP domain (see Additional file 10). Besides, another predicted putative effector, PteL, was detected in the supernatants of the WT strain, although its fold change was slightly below the predefined threshold (FC 3.552 instead of 4.0; Additional file 8). Due to its homology with the VopQ effector of *Vibrio parahaemolyticus* (see Additional file 9), we decided to include it in the subsequent analyses and hereafter refer to it as AopL.

In order to validate the mass spectrometry data and to quantify the levels of the candidate effectors in both the bacterial cells and their extracellular fraction, a split luciferase system was used. This method is based on a high-affinity complementation in which the 11-amino acid HiBiT tag at the C-terminus of the effector interacts with an 18-kDa LgBiT fragment. Upon addition of the furimazine substrate, the reconstituted luciferase complex produces luminescence, allowing accurate quantification of the tagged effector [49, 58]. Reporter strains of *A. schubertii* were constructed for each candidate effector by placing a HiBiT tag to the C-terminus of the effector in the WT, ΔAPI1, and ΔAPI2 genetic backgrounds. Luminescence measurements were used to determine the level of the candidate effector in both the intracellular and extracellular fractions under standard conditions and under low Ca^2+^/high Mg^2+^ concentrations. As shown in Figure 3C (absolute amounts of candidate effector) and Additional file 11 (extracellular fraction as a percentage of total), the majority of candidate effectors were exclusively secreted by the API1 injectisome after activation in low Ca^2+^/high Mg^2+^ conditions. Specifically, AopH, AopT, and AopU exhibited efficient secretion relative to their intracellular concentrations, indicating a highly efficient export. In contrast, AopO, AopI, AopJ, and AopL showed minimal secretion and were predominantly maintained intracellularly. Furthermore, despite the evaluation of two independent clones, expression of AopL^HiBiT^ was undetectable in the ΔAPI2 background for unclear reasons, preventing assessment of secretion via API2.

Together, these data show that the secretion mediated by the API1 injectisome of *A. schubertii* is artificially stimulated in vitro by low Ca^2+^/high Mg^2+^ concentration. Besides, we experimentally identified several predicted candidate effectors as well as two novel candidate effectors in *A. schubertii* ATCC 43700 that are secreted by the API1 injectisome.

### AopI, AopJ, AopL, AopT and AopU are novel effectors of *Aeromonas* species

To further corroborate the in vitro secretion results, we investigated whether the candidate effectors were successfully delivered into the host cells. The HiBiT-tagged effector reporter strains were used to infect HeLa cells expressing LgBiT with a MOI of 50:1, in the presence of a cell-permeable furimazine substrate. Successful translocation was demonstrated by an increase in luminescence (translocation signal) resulting from complementation of the HiBiT tag and the LgBiT subunit in the host cell cytosol [49, 58].

**Figure 4:**
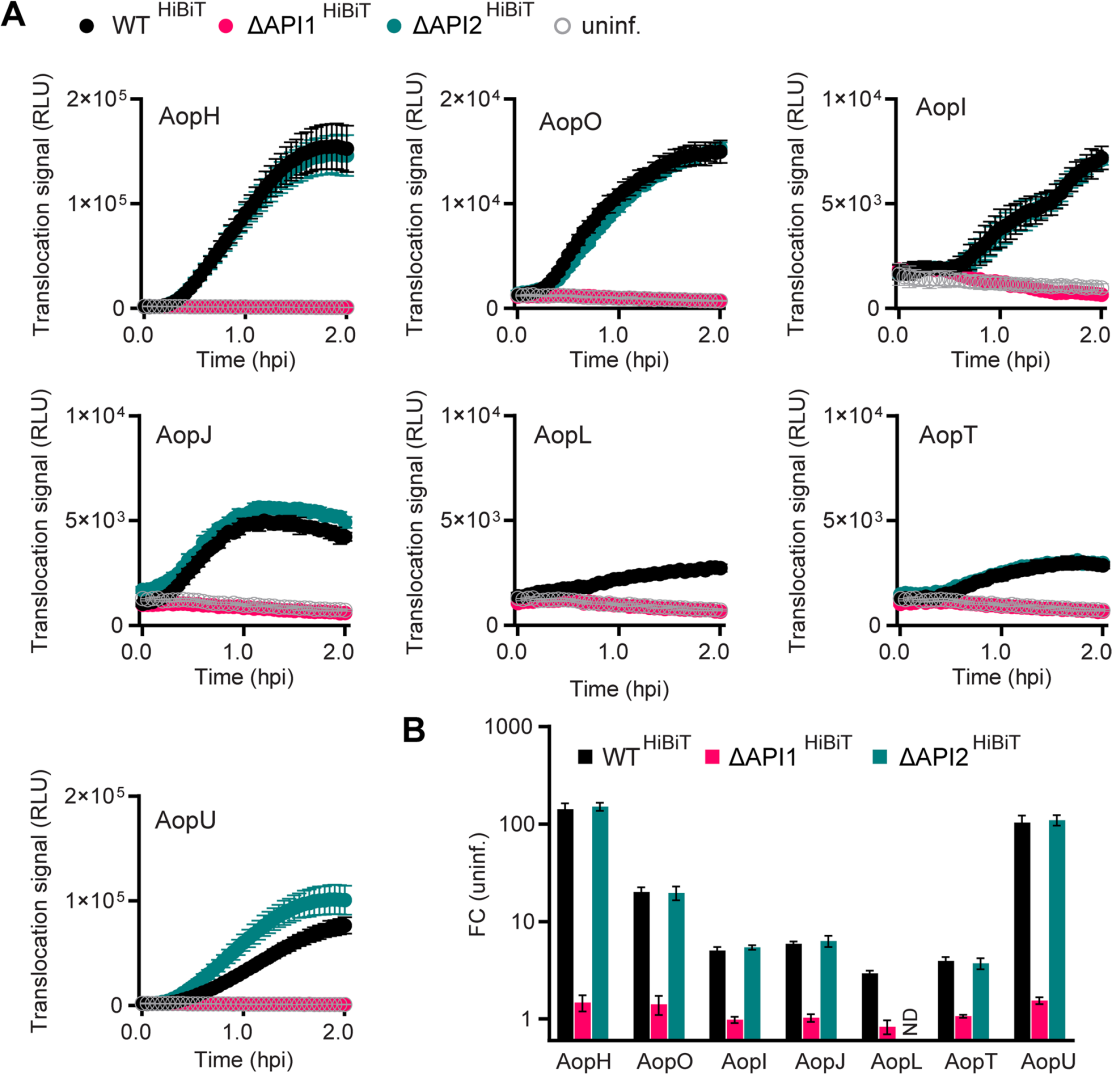
**AopI, AopJ, AopL, AopT and AopU represent novel effectors of *Aeromonas*** **species.** LgBit-expressing HeLa cells were infected with wild-type reporter strains (WT^HiBiT^) or mutant reporter strain derivatives lacking API1 (ΔAPI1^HiBiT^) or API2 (ΔAPI2^HiBiT^) injectisomes at MOI 50:1. Infections were performed in the presence of a cell-permeable furimazine substrate. Luminescence was measured every 3 min and expressed as relative luminescence units (RLU). **A** Data represent the mean ± SD of triplicate wells from a representative experiment out of three independent experiments. The candidate effector^HiBiT^ is indicated. ND, not determined for *aopL*^HiBiT^ /ΔAPI2. **B** Fold change of luminescence for cells infected with reporter strains relative to uninfected cells. Data represent the mean fold change in maximum luminescence ± SD, calculated from three independent experiments, each performed in triplicate.

As shown in Figure 4A, luminescence was observed within the first hour of infection for all seven candidate effectors of *A. schubertii* ATCC 43700, including previously characterized *A. salmonicida* effectors AopH and AopO as well as the newly identified effectors AopI, AopJ, AopL, AopT, and AopU. The translocation of candidate effectors was exclusively mediated by the API1 injectisome, with the exception of AopL, which could not be tested due to the undetectable expression of AopL^HiBiT^ in the ΔAPI2 background (as mentioned above). Interestingly, the magnitude of the translocation signal for the respective effectors (Figure 4B) did not consistently match their in vitro secretion levels. While both AopH and AopU exhibited efficient secretion and translocation, AopO showed an unexpectedly high translocation signal despite its low secretion in *A. schubertii* cultures. In contrast, AopT, which was efficiently secreted in vitro, showed a reduced translocation signal. The remaining effectors, namely AopJ, AopI, and AopL, showed translocation signals consistent with their in vitro secretion levels.

Overall, these results demonstrate that AopI, AopJ, AopL, AopT, and AopU represent novel effectors within the genus *Aeromonas*.

### Pro-survival effector AopI counteracts the cytotoxic effects of AopL in HeLa cells

**Figure 5:**
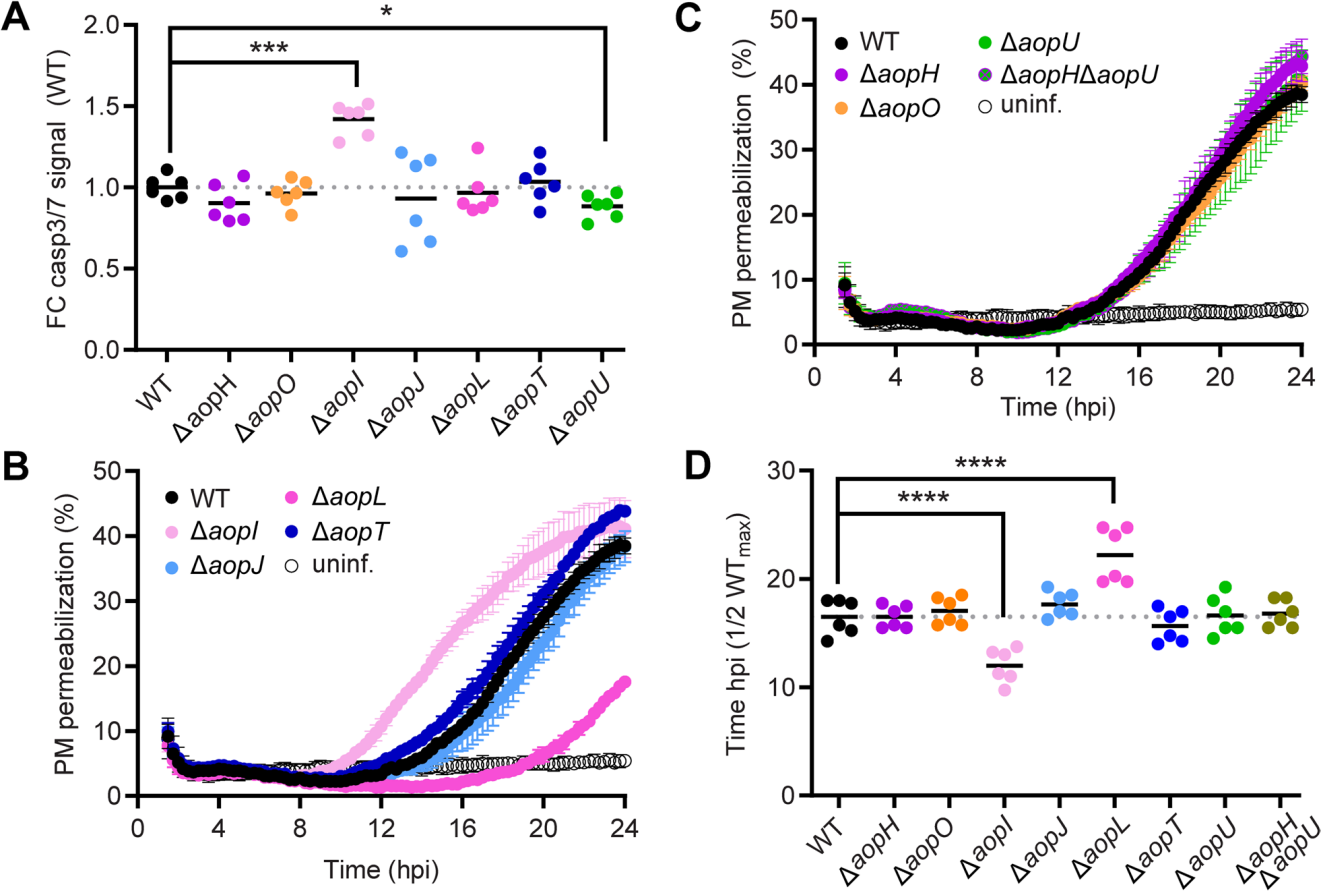
**Pro-survival effector AopI counteracts the cytotoxic effects of AopL in HeLa cells.** HeLa cells were either left uninfected or infected with *A. schubertii* wild-type (WT) or mutant strains lacking specific API1 effectors (Δ*aopH*, Δ*aopO*, Δ*aopI*, Δ*aopJ*, Δ*aopL*, Δ*aopT*, and Δ*aopU*) or their combination (Δ*aopH* Δ*aopU*) at MOI 10:1, as indicated. One hour post-infection, the extracellular bacteria were eliminated by the addition of gentamicin. **A** Caspase-3 and/or caspase-7 activation in infected HeLa cells was measured at 7 h post-infection using the Caspase-Glo 3/7 assay, which detects cleavage of a proluminescent caspase-3/7 substrate. Data are presented as the fold change (FC) of luminescence of cells infected with mutant strains relative to those infected with the wild-type strain. Data represent two independent experiments, each with triplicate wells. *p < 0.05, ***p < 0.001, unpaired two-tailed *t-*test. **B**–**C** Real-time kinetics of plasma membrane (PM) permeabilization of infected HeLa cells was measured using the fluorescent DNA-binding dye CellTox Green. PM permeabilization is expressed relative to complete permeabilization induced by a cell lysis solution. Data represent the mean ± SD of triplicate wells and are representative of 2 independent experiments. **D** Statistical analysis of PM permeabilization. Data are expressed as the time required to reach 50% of the maximal permeabilization observed in the WT-infected cells. Values represent two independent experiments, each performed in triplicate wells. ****p < 0.0001, unpaired two-tailed *t*-test.

To clarify the function of the newly identified effectors in the context of host cell cytotoxicity, individual effector genes were deleted from the *A. schubertii* genome, and the resulting mutant strains were examined for their effects on caspase activation and plasma membrane integrity. Remarkably, the majority of the mutant strains showed comparable characteristics to the WT strain, both in terms of caspase-3/7 activation at 7 h post-infection and overall impairment of plasma membrane integrity (Figures 5A–D). Even the double mutant Δ*aopH*/Δ*aopU*, which lacks the genes encoding the highly translocated effectors, did not significantly alter overall cytotoxicity (Figure 5C). However, the Δ*aopI* mutant showed a significant increase in caspase activity (Figure 5A), which corresponded with increased cellular necrosis at later time points during infection (Figures 5B, D). This finding suggests that AopI inhibits caspase-3/7 activation throughout the infection process and delays the overall cytotoxic effect of API1. In addition, the Δ*aopL* mutant showed a delay in plasma membrane permeabilization that occurred approximately 5 h later than during the infection with the WT strain (Figure 5D). This delay was not accompanied by a change in caspase-3/7 activation, suggesting that AopL promotes a necrotic pathway independent of caspase-3/7 activation. In contrast, the Δ*aopU* mutant exhibited a modest but statistically significant reduction in caspase-3/7 activation, however, this was not reflected in changes in overall necrosis (Figures 5A, C–D).

Altogether, these results highlight the essential role of AopL in inducing cell death, albeit via a mechanism that does not rely primarily on caspase-3/7 activation. In contrast, AopI acts as a pro-survival effector that counteracts the activation of cell death pathways during infection.

## Discussion

This study provides new insights into the function of the T3SS and its effector proteins in *A. schubertii*. Using a HeLa cell model, we demonstrated that the API1 injectisome is essential for the cytotoxic effects of *A.* *schubertii*, while API2 injectisome contributes minimally in this model system. This distinction is consistent with previous observations that bacterial genomes often harbor multiple T3SS loci, each with specialized functions.

API1 injectisome of *A. schubertii* belongs to the Ysc family of injectisomes, which also includes injectisomes of *Yersinia* spp., *Pseudomonas aeruginosa*, and *Vibrio* spp. [16]. Our findings indicate that API1 secretion of *A. schubertii* can be activated by artificial cues, particularly low Ca^2+^ and high Mg^2+^ concentrations, which is likely mediated by both transcriptional and posttranslational mechanisms. Similar to *P. aeruginosa*, API1 encodes components of the ExsACDE regulatory circuit. In *P. aeruginosa*, expression of the T3SS is regulated by ExsA, a transcriptional activator that is bound to a negative regulator ExsD under non-permissive conditions. Upon exposure to inducing signals, such as contact with host cells or low Ca^2+^ level, ExsC sequesters ExsD, thereby releasing ExsA to initiate T3SS gene expression [59, 60]. In addition, low Ca^2+^ acts as an artificial activation signal by triggering the release of the gatekeeper protein from the SctV component in various injectisomes, including those of *Yersinia* and *Bordetella*, which subsequently allows for the secretion of effectors in the so-called second substrate switch event [49, 57, 61]. High Mg^2+^ concentrations, on the other hand, have been reported to inhibit the two-component PhoPQ system, thereby enhancing the transcription of the API1 machinery [56]. However, the exact roles of low Ca^2+^ and high Mg^2+^ concentrations in the activation of the API1 injectisome of *A. schubertii* remain to be further established.

By depleting Ca^2+^ and increasing Mg^2+^ concentration to artificially activate the API1 injectisome, we identified seven candidate effectors by mass spectrometry. Of these, two (AopH and AopO) show homology with effector proteins from *A. salmonicida* [26], three (AopI, AopJ, and AopL) were predicted in a recent bioinformatic analysis [35], and two (AopT and AopU) are novel. Interestingly, our mass spectrometry analysis did not identify all bioinformatically predicted API1 T3SS effectors [35], including PteA, which is homologous to the cytotoxic effector BteA of *Bordetella* species [62, 63]. Indeed, *pteA* gene in *A. schubertii* ATCC 43700 is not pseudogenized, and a T3SS chaperone is located upstream of its coding region, supporting its classification as a T3SS effector. This suggests that alternative induction conditions, such as growth at 37 °C or deletion of the ExsD negative regulator, may reveal additional T3SS effectors of the API1 injectisome*.*

To detect the translocation of the identified candidate effector into host cells, we used a split luciferase system [49, 58]. Intriguingly, the translocation signals did not consistently correlate with the observed intracellular abundance or secretion levels in vitro. For example, AopO showed an unexpectedly high translocation signal, suggesting the involvement of additional regulatory factors that facilitate its preferential delivery or enhance expression after direct contact with a host cell. Alternatively, variations in HiBiT-LgBit complementation efficiency between bacterial supernatants and the host cell cytosol may have played a role, possibly influenced by the accessibility of the HiBiT tag to the LgBit subunit.

**Figure 6:**
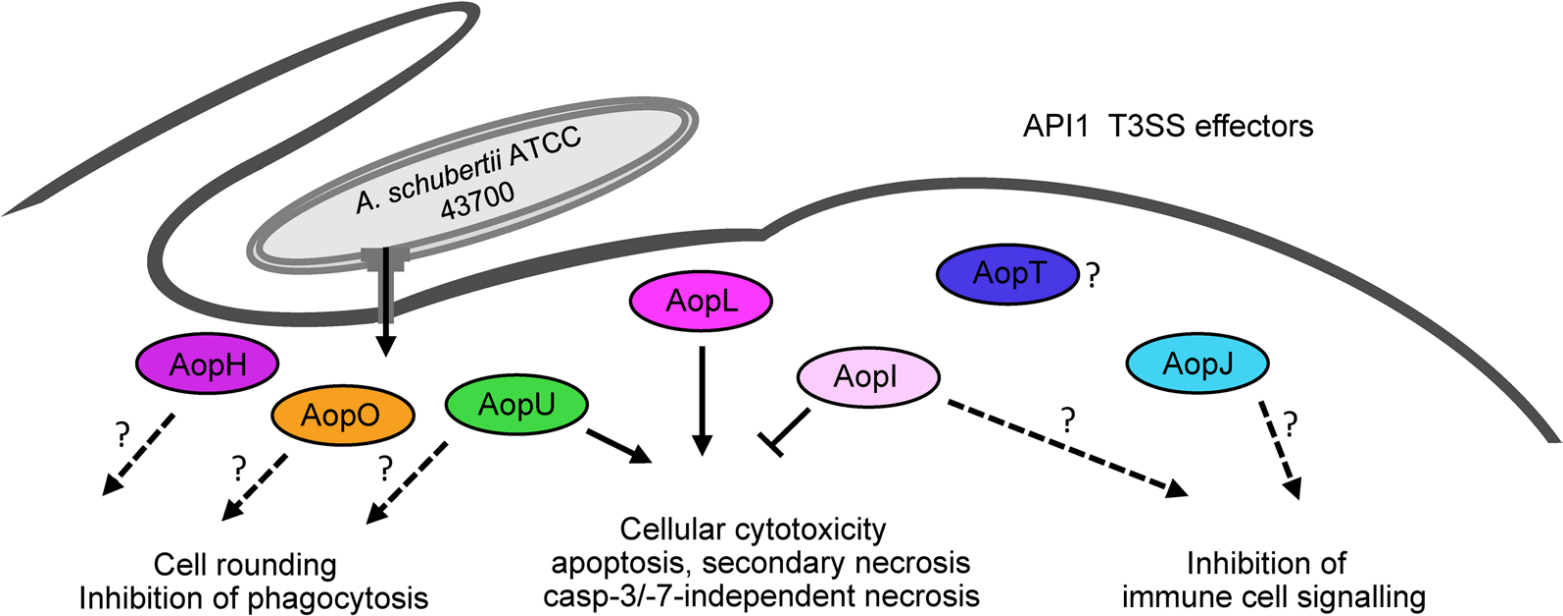
**Proposed model of T3SS effector actions delivered by the API1 injectisome in** ***A. schubertii.*** During interaction with host cells, *A. schubertii* translocates several T3SS effectors into the host cytosol via the API1 injectosome. AopL induces caspase-3/-7-independent necrosis, possibly by targeting the lysosomal V-ATPase, a prediction supported by its homology with VopQ from *Vibrio parahaemolyticus*. In contrast, AopU promotes caspase-3/-7-dependent apoptosis. These cytotoxic effects are counteracted by AopI, a pro-survival effector, which, based on its structural similarity to *P. aeruginosa* ExoY, is presumed to act as a nucleotidyl cyclase. Sequence homology and domain analysis also suggest that AopU, AopH, and AopO induce cell rounding and inhibit phagocytosis, while AopI and AopJ may interfere with host immune signaling pathways. The specific role of AopT remains uncertain.

Although the observed cytotoxicity was dependent on the API1 injectisome, cell death was not completely prevented by the absence of individual API1 effectors. This observation suggests a redundancy or functional overlap between API1 effectors, which is quite common for T3SS effectors [64]. As depicted in Figure 6, of the 7 identified effectors, only AopL and AopI were critical modulators of HeLa cell cytotoxicity, although in a contradictory manner. Specifically, AopL accelerated necrosis via a mechanism independent of caspase-3/7, mirroring the action of its homolog VopQ in *V. parahaemolyticus*. This effector targets the host cell lysosomal V-ATPase and triggers several deleterious effects, including lysosomal deacidification, disruption of redox homeostasis, and induction of autophagy and cell necrosis [65–68]. In contrast, AopI showed a protective, pro-survival effect by inhibiting the activation of caspase-3/7 and postponing overall cellular cytotoxicity. This protein shows homology to ExoY of *P. aeruginosa*, which is a nucleotidyl cyclase that is activated by filamentous actin [69]. Interestingly, ExoY has been shown to modulate innate immune responses as well as to protect against cytotoxicity induced by *P. aeruginosa* in human bronchial epithelial cells [70, 71].

In addition to these key modulators, AopU also played a minor yet significant role in the activation of caspase-3/7, although its precise function is unclear (Figure 6). AopU is predicted to inactivate Rho, Rac, and/or Cdc42 by GTP hydrolysis mediated by its putative RhoGAP activity, similar to *Aeromonas* AexT/AexU [34, 72]. This putative activity is consistent with the early morphological changes observed during *A. schubertii* infection, which were characterized by rounding of cells, likely due to disruption of the cell cytoskeleton and/or loss of adhesion. However, in contrast to AexT/AexU, AopU does not have an ADP-ribosyltransferase domain. In addition, AopH, a putative phosphotyrosine phosphatase, and AopO, a putative serine/threonine kinase, may also play a role in these morphological changes, as indicated in Figure 6. Their *Yersinia* homologs, YopH and YopO, are known for their ability to alter the actin cytoskeleton, inhibit phagocytosis, and disrupt focal adhesions [26]. Furthermore, among the newly identified effectors, AopJ may play a role in disrupting innate immune signaling due to its homology with a family of phosphothreonine lyase effectors, including *Shigella* OspF and *Salmonella* SpvC, which inhibit MAPK signaling pathways and downregulate inflammatory cytokine production [73, 74]. At the same time, the function of AopT, a putative lipase, remains unknown (Figure 6).

However, it should be mentioned that although HeLa cells are a widely used model in host–pathogen interaction studies, they may not fully replicate the interactions between *A. schubertii* and fish host cells. In the future, it will be important to investigate how the identified effectors affect cytotoxicity in fish epithelial cells, impair macrophage phagocytic activity, alter susceptibility to predation by amoebae, and contribute to the subversion of innate or adaptive immune responses. Given the increasing threat that *A. schubertii* poses to aquaculture of snakehead fish, it will also be critical to assess whether the T3SS and its effectors contribute to the cytotoxicity and formation of white nodules of affected fish, as well as to overall fish morbidity [42, 43, 75]. Additionally, sequencing of more strains of *A. schubertii* will be important to better understand the relationship between virulence and the presence of API1 or API2 loci and/or specific subsets of effectors. Currently, only seven entries of *A. schubertii* genomes are available on NCBI, with *A. schubertii* ATCC 43700 (NCBI RefSeq: GCF_001481395.1) and CECT4240 (NCBI RefSeq GCF_000820105.1) representing the same isolate, originally obtained from a human forehead abscess in Texas, United States. Interestingly, all available genomes of *A. schubertii* harbor the API1 locus, and all except CHULA2021b strain contain the API2 locus. However, as shown in Additional file 12, the distribution of identified API1 T3SS effectors is more variable: AopL and AopT are present in all isolates; AopH, AopO, and AopJ are found in all but one isolate; AopU is absent in two isolates; and AopI is detected only in two isolates.

In our work, all identified T3SS effectors, with the exception of AopL, which could not be tested, were specifically translocated into the host cell via the API1 injectisome. This specificity could be due to the intrinsic selectivity of the API1 injectisome or the inactivity of the API2 injectisome during infection of HeLa cells. API2 injectisome shares homology with the injectisome of *Salmonella enterica*, which belongs to the Ssa-Esc family and is activated after internalization of the bacterium into acidic compartments by low pH [76]. Although bacteria of the *Aeromonas* genus are predominantly classified as extracellular, some reports have shown that *Aeromonas* can invade epithelial cells and persist within amoebae [77–80]. Therefore, it also remains important to determine whether the API2 injectisome acts extracellularly or is activated only after bacterial uptake, possibly allowing for intracellular persistence.

In conclusion, our results emphasize the parallels between the API1 effectors of *A. schubertii* and the T3SS effectors of other Gram-negative bacteria, particularly *A. salmonicida*, *P. aeruginosa*, and *V. parahaemolyticus*, which supports the idea of horizontal gene transfer [10–12]. However, it is important to mention that the identified repertoire of API1 effectors is unique for *A. schubertii* [35]. This suggests that *A. schubertii* may have acquired two distinct API injectisomes and their effectors through horizontal gene transfer, and retained them for a competitive advantage. As such, our study improves the understanding of *A. schubertii*-mediated cytotoxicity and provides experimental identification of novel *Aeromonas* effectors.

## Supplementary Information

**Additional file 1. List of bacterial strains used in this study.**

**Additional file 2. List of plasmids used in this study.**

** Additional file 3. List of PCR primers used for plasmid construction via Gibson assembly in this study. **Uppercase letters represent the PCR primer regions corresponding to chromosomal DNA of *A. schubertii* ATCC 43700, while lowercase letters in H1-fw and H2-rv indicate sequences homologous to the linearized pAX2 vector. Italic underlined letters in H1-rv and H2-fw correspond to sequences inserted into the plasmid, including the GSSG linker followed by HiBit-tag, as required.

** Additional file 4. Accession numbers of SctN and SctC proteins used for phylogenetic analysis.**

** Additional file 5. Phylogenetic analysis of core proteins in API1 and API2 injectisomes. **To analyze the phylogenetic relationships of SctN and SctC proteins from *Aeromonas *API1 and API2, minimum evolution trees based on *p*-distance were constructed using MEGA11 software. Protein sequences were compared to their orthologs in different bacterial species. The results indicate that AscN and AscC of API1 map within the Ysc family, named after *Yersinia* spp., while SctN and SctC of API2 belong to the SsaEsc family, which is characteristic for the SPI2-encoded T3SS injectisome in *Salmonella enterica* serovar Typhimurium.

** Additional file 6. Imaging of morphological changes in HeLa cells. **HeLa cells were either left uninfected or infected with *A. schubertii* wild-type (WT) or mutant strains lacking the API1 (ΔAPI1) or API2 (ΔAPI2) injectisomes, due to the deletion of the respective T3SS ATPases, at MOI of 10:1. One hour post-infection, the extracellular bacteria were eliminated by addition of gentamicin, and morphological changes in HeLa cell were recorded as a time-lapse of 24 hours with frame intervals of 10 minutes. Scale bar, 20 µm.

** Additional file 7. Western blot analysis of caspase-3 activation. **HeLa cells were either left uninfected or infected with *A. schubertii* wild-type (WT) or mutant strains lacking the API1 (ΔAPI1) or API2 (ΔAPI2) injectisomes, at an MOI of 10:1. One hour post-infection, the extracellular bacteria were eliminated by the addition of gentamicin. Whole-cell lysates were prepared at indicated time points, separated by SDS-PAGE, and analyzed by immunoblotting using an antibody that detects both the full-length inactive form of caspase-3 (pro-casp 3, 35 kDa) and the cleaved caspase-3 fragment (cleaved casp 3, 17 kDa). βactin (40 kDa) was used as a loading control. HeLa cells treated with 1 µM staurosporine (STS) served as a positive control. Data are representative of 2 independent experiments.

**Additional file 8. Significant changes in the secretome of WT strain compared to the ΔAPI1 derivative in TSB supplemented with 0.5 mM EGTA and 20 mM MgCl**_**2**_**.**This table contains Log_2_-transformed LFQ proteomic data. Significant changes were defined as |fold change| ≥ 4 and -log_10_*P* value ≥ 2, corresponding to *P* ≤ 0.01.

** Additional file 9. Sequence alignment of AopI, AopJ, and AopL with homologous T3SS effectors. **ExoY from *Pseudomonas aeruginosa* (WP_003115517.1) in (A), OspF from *Shigella flexneri* (HCR8314926.1) in (B), and VopQ from *Vibrio parahaemolyticus* (WP_005464333.1) in (C) were used for comparison. Amino acid sequences were aligned using the Clustal Omega online alignment tool available at the Uniprot website and visualized with the percentage identity scheme in Jalview.

**Additional file 10. Candidate effectors of the API1 injectisome.** Amino acid sequences are provided.

**Additional file 11. Response of candidate effector proteins to low** **Ca**^**2+**^**/ high Mg**^**2+**^ **concentrations.** Cultures of WT reporter strains (WT^HiBiT^) and mutant derivatives lacking API1 (ΔAPI1^HiBiT^) or API2 (ΔAPI2^HiBiT^) injectisomes were grown in TSB medium without and with supplementation of 0.5 mM EGTA and 20 mM MgCl_2_. The amount of candidate effector^HiBiT^ in each fraction was expressed as a percentage of the total candidate effectorHiBiT present in the culture. Data are representative of 3 independent experiments. ND, not determined.

**Additional file 12. Distribution and similarity of the identified T3SS effectors across genomes of** ***A. schubertii*** **strains.** The heatmap displays the presence and percentage similarity of T3SS effectors identified in *A. schubertii* ATCC 43700 across other available *A. schubertii* genomes. Absence of an effector is indicated by black / ND (not detected). The following genomes were analyzed: the type strain *A. schubertii* ATCC 43700 (NCBI RefSeq: GCF_001481395.1), originally isolated from a forehead abscess in Texas, United States. Alternative designations for this strain include CECT4240, CDC 2446-81, CCTM La 3016, CCUG 27820, DSM 4882, JCM 7373, LMG 9074, and NCIMB 13161. A different passage of this strain is also deposited in NCBI under its alternative name CECT4240 (NCBI RefSeq GCF_000820105.1). Strain A40 (NCBI RefSeq: GCF_045983075.1) was isolated from a case of *Aeromonas* septicemia in Asian sea bass (*Lates calcarifer*) in Thailand. Strains CHULA2021a (NCBI RefSeq: GCF_020089825.1) and CHULA2021b (NCBI RefSeq: GCF_020089835.1) were recovered from mass mortality events in Asian sea bass (*Lates calcarifer*) in Thailand. Additionally, strain LF1708 (NCBI RefSeq: GCF_004919485.1) was isolated from diseased Nile tilapia (*Oreochromis niloticus*), whereas strain WL1483 (NCBI RefSeq: GCF_001447335.1) was isolated from diseased snakehead fish *(Channa argus*), both in China.

## Data Availability

The mass spectrometry proteomics data have been deposited to the ProteomeXchange Consortium via the PRIDE [52] partner repository with the dataset identifier PXD062075. All other data necessary to validate the conclusions of this study are included in this published article and its additional files.
