# Supplementary material for: Cytotoxicity induced by Aeromonas schubertii is orchestrated by a unique set of type III secretion system effectors
Source: Vet Res. 2025 Jun 8;56:113. doi: 10.1186/s13567-025-01548-2 (PMC12147276; doi:10.1186/s13567-025-01548-2)
Supplement: Supplementary file 1 — Additional file 1. List of bacterial strains used in this study. [file 13567_2025_1548_MOESM1_ESM.pdf]

Additional file 1. List of bacterial strains used in this study.

Strain	Genotype and relevant description	Int. no.	Reference
***E. coli* strains**			
XL1-Blue	*rec*A1 *end*A1 *gyr*A96 *thi*-1 *hsd*R17 *sup*E44 *rel*A1 *lac* F´ *pro*AB *lacIq*ZΔM15 Tn10 Tet^r		
SM10 λpir	*thi thr leu ton*A *lac*Y *sup*E *rec*A::RP4-2-Tc::Mu Km λpir		
***A. schubertii* ATCC 43700 strains**			
WT	*Asch* WT; wild type *Aeromonas schubertii* ATCC 43700, source: Czech Collection of Microorganisms, Masaryk University, Brno, Czech Republic	A002	cat.no. # CCM 4356T
ΔAPI1	*Asch* ΔAPI1; *Asch* WT strain derivative with API1 *sctN* ATPase in-frame deletion of codons K7-L438	A021	This study
ΔAPI2	*Asch* ΔAPI2; *Asch* WT strain derivative with API2 *sctN* ATPase in-frame deletion of codons H7-G435	A026	This study
aopH^{HiBiT}	*Asch* WT encoding *aopH* allele fused to a GSSG linker and a HiBiT-tag at the C-terminus	A144	This study
aopH^{HiBiT} / ΔAPI1	*aopH*^{HiBiT} derivative with API1 *sctN* ATPase in-frame deletion of codons K7-L438	A146	This study
aopH^{HiBiT} / ΔAPI2	*aopH*^{HiBiT} derivative with API2 *sctN* ATPase in-frame deletion of codons H7-G435	A147	This study
aopO^{HiBiT}	*Asch* WT encoding *aopO* allele fused to a GSSG linker and a HiBiT-tag at the C-terminus	A151	This study
aopO^{HiBiT} / ΔAPI1	*aopO*^{HiBiT} derivative with API1 *sctN* ATPase in-frame deletion of codons K7-L438	A153	This study
aopO^{HiBiT} / ΔAPI2	*aopO*^{HiBiT} derivative with API2 *sctN* ATPase in-frame deletion of codons H7-G435	A155	This study
aopI^{HiBiT}	*Asch* WT encoding *aopI* allele fused to a GSSG linker and a HiBiT-tag at the C-terminus	A076	This study
aopI^{HiBiT} / ΔAPI1	*aopI* ^{HiBiT} derivative with API1 *sctN* ATPase in-frame deletion of codons K7-L438	A079	This study
aopI^{HiBiT} / ΔAPI2	*aopI* ^{HiBiT} derivative with API2 *sctN* ATPase in-frame deletion of codons H7-G435	A082	This study
aopJ^{HiBiT}	*Asch* WT encoding *aopJ* allele fused to a GSSG linker and a HiBiT-tag at the C-terminus	A109	This study
aopJ^{HiBiT} / ΔAPI1	*aopJ* ^{HiBiT} derivative with API1 *sctN* ATPase in-frame deletion of codons K7-L438	A110	This study
aopJ^{HiBiT} / ΔAPI2	*aopJ* ^{HiBiT} derivative with API2 *sctN* ATPase in-frame deletion of codons H7-G435	A113	This study
aopL^{HiBiT}	*Asch* WT encoding *aopL* allele fused to a GSSG linker and a HiBiT-tag at the C-terminus; two clones from different merodiploids	A130<br>A180	This study
aopL^{HiBiT} / ΔAPI1	*aopL* ^{HiBiT} derivative with API1 *sctN* ATPase in-frame deletion of codons K7-L438	A131	This study
aopL^{HiBiT} / ΔAPI2	*aopL* ^{HiBiT} derivative with API2 *sctN* ATPase in-frame deletion of codons H7-G435; two clones from different merodiploids	A132<br>A182	This study
aopT^{HiBiT}	*Asch* WT encoding *aopT* allele fused to a GSSG linker and a HiBiT-tag at the C-terminus	A143	This study
aopT^{HiBiT} / ΔAPI1	*aopT* ^{HiBiT} derivative with API1 *sctN* ATPase in-frame deletion of codons K7-L438	A139	This study
aopT^{HiBiT} / ΔAPI2	*aopT* ^{HiBiT} derivative with API2 *sctN* ATPase in-frame deletion of codons H7-G435	A141	This study

aopU^{HiBiT}	*Asch* WT encoding *aopU* allele fused to a GSSG linker and a HiBiT-tag at the C-terminus	A115	This study
aopU^{HiBiT} / ΔAPI1	*aopU* ^{HiBiT} derivative with API1 *sctN* ATPase in-frame deletion of codons K7-L438	A118	This study
aopU^{HiBiT} / ΔAPI2	*aopU* ^{HiBiT} derivative with API2 *sctN* ATPase in-frame deletion of codons H7-G435	A121	This study
Δ*aopH*	*Asch ΔaopH*; *Asch* WT strain derivative with *aopH* allele in-frame deletion of codons S4-L443	A100	This study
Δ*aopO*	*Asch ΔaopO*; *Asch* WT strain derivative with *aopO* allele in-frame deletion of codons I4-W726	A103	This study
Δ*aopI*	*Asch ΔAopI*; *Asch* WT strain derivative with *AopI* allele in-frame deletion of codons D4-G374	A029	This study
Δ*aopJ*	*Asch ΔptJI*; *Asch* WT strain derivative with *aopJ* allele in-frame deletion of codons K4-L233	A091	This study
Δ*aopL*	*Asch ΔaopL*; *Asch* WT strain derivative with *aopL* allele in-frame deletion of codons S4-G479	A097<br>A098<br>A099	This study
Δ*aopT*	*Asch ΔaopT*; *Asch* WT strain derivative with *aopT* allele in-frame deletion of codons N4-I354	A172	This study
Δ*aopU*	*Asch ΔaopU*; *Asch* WT strain derivative with *aopU* allele in-frame deletion of codons A5-Q257	A096	This study
Δ*aopH /* Δ*aopU*	*Asch ΔaopH/ΔaopU*; *Asch ΔaopH* strain derivative with *aopU* allele in-frame deletion of codons N4-I354	A177	This study
