# Supplementary material for: Cytotoxicity induced by Aeromonas schubertii is orchestrated by a unique set of type III secretion system effectors
Source: Vet Res. 2025 Jun 8;56:113. doi: 10.1186/s13567-025-01548-2 (PMC12147276; doi:10.1186/s13567-025-01548-2)
Supplement: Supplementary file 2 — Additional file 2. List of plasmids used in this study. [file 13567_2025_1548_MOESM2_ESM.pdf]

Additional file 2. List of plasmids used in this study.

Plasmid	Description	Reference
pAX2	allelic exchange vector with GFP merodiploid tracker, temperature-sensitive origin of replication, and TetR-controlled kill switch	Addgene #117398
pAX2-ΔAPI1	pAX2 vector containing *Asch* ATCC 43700 homology regions h1 (643 bp) and h2 (571 bp) flanking an in-frame deletion of codons K7-L438 of the T3SS ATPase *sctN* within API1	This study
pAX2-ΔAPI2	pAX2 vector containing Asch ATCC 43700 homology regions h1 (630 bp) and h2 (654 bp) flanking an in-frame deletion of codons H7-G435 of the T3SS ATPase sctN within API2	This study
pAX2-*aopH*^{HiBiT}	pAX2 vector containing homology regions h1 (610 bp) and h2 (715 bp) flanking the C-terminus of the *aopH* allele of Asch ATCC 43700, with an intervening insertion of codons coding for a GSSG linker followed by a HiBiT-tag	This study
pAX2-*aopO*^{HiBiT}	pAX2 vector containing homology regions h1 (612 bp) and h2 (600 bp) flanking the C-terminus of the *aopH* allele of Asch ATCC 43700, with an intervening insertion of codons coding for a GSSG linker followed by a HiBiT-tag	This study
pAX2-*aopI*^{HiBiT}	pAX2 vector containing homology regions h1 (617 bp) and h2 (543 bp) flanking the C-terminus of the *aopH* allele of Asch ATCC 43700, with an intervening insertion of codons coding for a GSSG linker followed by a HiBiT-tag	This study
pAX2-*aopJ*^{HiBiT}	pAX2 vector containing homology regions h1 (716 bp) and h2 (777 bp) flanking the C-terminus of the *aopH* allele of Asch ATCC 43700, with an intervening insertion of codons coding for a GSSG linker followed by a HiBiT-tag	This study
pAX2-*aopL*^{HiBiT}	pAX2 vector containing homology regions h1 (623 bp) and h2 (529 bp) flanking the C-terminus of the *aopH* allele of Asch ATCC 43700, with an intervening insertion of codons coding for a GSSG linker followed by a HiBiT-tag	This study
pAX2-*aopT*^{HiBiT}	pAX2 vector containing homology regions h1 (624 bp) and h2 (621 bp) flanking the C-terminus of the *aopH* allele of Asch ATCC 43700, with an intervening insertion of codons coding for a GSSG linker followed by a HiBiT-tag	This study
pAX2-*aopU*^{HiBiT}	pAX2 vector containing homology regions h1 (499 bp) and h2 (746 bp) flanking the C-terminus of the *aopH* allele of Asch ATCC 43700, with an intervening insertion of codons coding for a GSSG linker followed by a HiBiT-tag	This study
pAX2-Δ*aopH*	pAX2 vector containing *Asch* ATCC 43700 homology regions h1 (694 bp) and h2 (721 bp) flanking an in-frame deletion of codons S4-L443 of *aopH* allele	This study
pAX2-Δ*aopO*	pAX2 vector containing *Asch* ATCC 43700 homology regions h1 (758 bp) and h2 (606 bp) flanking an in-frame deletion of codons I4-W726	This study
pAX2-Δ*aopI*	pAX2 vector containing *Asch* ATCC 43700 homology regions h1 (594 bp) and h2 (685 bp) flanking an in-frame deletion of codons D4-G374	This study
pAX2-Δ*aopJ*	pAX2 vector containing *Asch* ATCC 43700 homology regions h1 (833 bp) and h2 (783 bp) flanking an in-frame deletion of codons K4-L233	This study
pAX2-Δ*aopL*	pAX2 vector containing *Asch* ATCC 43700 homology regions h1 (796 bp) and h2 (859 bp) flanking an in-frame deletion of codons S4-G479	This study
pAX2-Δ*aopT*	pAX2 vector containing *Asch* ATCC 43700 homology regions h1 (703 bp) and h2 (627 bp) flanking an in-frame deletion of codons N4-I354	This study
pAX2-Δ*aopU*	pAX2 vector containing *Asch* ATCC 43700 homology regions h1 (653 bp) and h2 (749 bp) flanking an in-frame deletion of codons A5-Q257	This study
