# Supplementary material for: Cytotoxicity induced by Aeromonas schubertii is orchestrated by a unique set of type III secretion system effectors
Source: Vet Res. 2025 Jun 8;56:113. doi: 10.1186/s13567-025-01548-2 (PMC12147276; doi:10.1186/s13567-025-01548-2)
Supplement: Supplementary file 3 — Additional file 3. List of PCR primers used for plasmid construction via Gibson assembly in this study. Uppercase letters represent the PCR primer regions corresponding to chromosomal DNA of A. schubertii ATCC 43700, while lowercase letters in H1-fw and H2-rv indicate sequences homologous to the linearized pAX2 vector. Italic underlined letters in H1-rv and H2-fw correspond to sequences inserted into the plasmid, including the GSSG linker followed by HiBit-tag, as required. [file 13567_2025_1548_MOESM3_ESM.pdf]

Additional file 3. List of PCR primers used for plasmid construction via Gibson assembly in this study. Uppercase letters represent the PCR primer regions corresponding to chromosomal DNA of *A. schubertii* ATCC 43700, while lowercase letters in H1-fw and H2-rv indicate sequences homologous to the linearized pAX2 vector. Italic underlined letters in H1-rv and H2-fw correspond to sequences inserted into the plasmid, including the GSSG linker followed by HiBit-tag, as required.

Gene	Mutation	Region	Sequence
ascN	Deletion ΔAPI1	HR1-fw	taggagtcgcgagtttaaacCTTGATAGCATCGCGCAGCTCG
		HR1-rv	GCGGATCATCGGGT*GGATCC*GTCGAGTGTGTGTGCCATGACATC
		HR2-fw	CACACACACTCGAC*GGATCC*ACCCGATGATCCGCCGGC
		HR2-rv	atttctcgagtctggtcgacCATCGCCTCGTCAAACTGGCG
sctN	Deletion ΔAPI2	HR1-fw	taggagtcgcgagtttaaacTCGCCCTCGACCGCTACG
		HR1-rv	TCTCAGCATGGGAG*GGATCC*ATAATCAGGCAGCCGCATGGGTC
		HR2-fw	GGCTGCCTGATTAT*GGATCC*CTCCCATGCTGAGACAGTTACTCG
		HR2-rv	atttctcgagtctggtcgacCCTGCGTTGATCATCAGGCCTG
aopH	Deletion	HR1-fw	gctaggagtcgcgagtttaaaccccGACAGCCTGGGGGTCTAATC
		HR1-rv	CTGTCACTTAGGCACG*GGATCC*TAATGCCATATGAACCTCAGCTTATGTT
		HR2-fw	TTCATATGGCATTA*GGATCC*CGTGCCTAAGTGACAGGAGAAGA
		HR2-rv	gcatttctcgagtctggtcgaccccTATCGTCGGTTCACTCTTTATCGC
	HiBit tagging	HR1-fw	gagtcgcgagtttaaaccccTGAGAGCCACATGCAGATGCT
		HR1-rv	*CAGCCGCCAGCCGCTCACTCCACTGGATCC*GGCACGCAACAAAGAACGCC
		HR2-fw	A*GTGAGCGGCTGGCGGCTGTTCAAGAAGATTAGC*TAAGTGACAGGAGAAGAGGCCAC
		HR2-rv	gcatttctcgagtctggtcgaccccTATCGTCGGTTCACTCTTTATCGC
aopO	Deletion	HR1-fw	gctaggagtcgcgagtttaaaccccGATCAATCCGGACGGGCTCTC
		HR1-rv	ATCGACTAGCGGAT*GGATCC*GATCTTCATGATGAATTACACCCAGCTTG
		HR2-fw	GTAATTCATCATGAAGATC*GGATCC*ATCCGCTAGTCGATGCCCAC
		HR2-rv	gcatttctcgagtctggtcgaccccCTCTGGGCCGCCTTGATGATATC
	HiBit tagging	HR1-fw	gagtcgcgagtttaaaccccCAGCTGCGCGGCAATATCAT
		HR1-rv	*AACAGCCGCCAGCCGCTCACTCCACTGGATCC*GCGGATCCACTCCTGGGA
		HR2-fw	*GTGAGCGGCTGGCGGCTGTTCAAGAAGATTAGC*TAGTCGATGCCCACAGACCC
		HR2-rv	gcatttctcgagtctggtcgaccccCTCTGGGCCGCCTTGATGATATC
aopI	Deletion	HR1-fw	gtcgcgagtttaaaccccGATCAATTAAATATCCTTCCCATCAATTAACA
		HR1-rv	TCGACTCACACCTT*GGATCC*GATACGCATCCTGACTCTCCTCT
		HR2-fw	TCAGGATGCGTATC*GGATCC*AAGGTGTGAGTCGATAATCGAGC
		HR2-rv	tctcgagtctggtcgaccccccGAGAGCGCTGCTGTTGTC
	HiBit tagging	HR1-fw	gagtcgcgagtttaaaccccACACCTATGAGTTTGTCGCCCG
		HR1-rv	*CTTCTTGAACAGCCGCCAGCCGCTCACTCCACTGGATCC*CACCTTGCCGCGAAAGAAGG
		HR2-fw	*GCTGGCGGCTGTTCAAGAAGATTAGC*TGAGTCGATAATCGAGCAGGGAT
		HR2-rv	tctcgagtctggtcgaccccGTAGAGATGGCTCAGGCCGATG

aopJ	Deletion	HR1-fw	gctaggagtcgcgagtttaaaccccGCGGTAGGATTCAAGCAGCAT
		HR1-rv	ACTGAGCCGGGTTATTCGGT*GGATCC*GGCTTTCATGGTGTGCTCCT
		HR2-fw	AGGAGCACACCATGAAAGCC*GGATCC*ACCGAATAACCCGGCTCAGT
		HR2-rv	gcatttctcgagtctggtcgaccccGTGCTGGTGCTCACGCTG
	HiBit tagging	HR1-fw	gagtcgcgagtttaaaccccAGGAGCACACCATGAAAGCC
		HR1-rv	*AACAGCCGCCAGCCGCTCACTCCACTGGATCC*TTCGGTCAGCAGGCGGTA
		HR2-fw	*GAGCGGCTGGCGGCTGTTCAAGAAGATTAGC*TAACCCGGCTCAGTGGGAG
		HR2-rv	gcatttctcgagtctggtcgaccccGTGCTGGTGCTCACGCTG
aopL	Deletion	HR1-fw	gctaggagtcgcgagtttaaaccccCTGAGCTTAGTGCATCGTTGGC
		HR1-rv	TTTTTTATAGTGCAGGCTAGAGCCA*GGATCC*GGTGGGCATGGGACTCTCC
		HR2-fw	GAGAGTCCCATGCCCACC*GGATCC*TGGCTCTAGCCTGCACTATAAAAAAC
		HR2-rv	gcatttctcgagtctggtcgaccccCGCCAAGCGATCTGATTGCC
	HiBit tagging	HR1-fw	gagtcgcgagtttaaaccccCCATGGTTGCCAGCAAGGC
		HR1-rv	*AACAGCCGCCAGCCGCTCACTCCACTGGATCC*GAGCCAGCCCTCCGCCA
		HR2-fw	*GTGAGCGGCTGGCGGCTGTTCAAGAAGATTAGC*TAGCCTGCACTATAAAAAACGCCAG
		HR2-rv	tctcgagtctggtcgaccccCGTTCGCCTTCCTGGAGCC
aopT	Deletion	HR1-fw	gagtcgcgagtttaaaccccCTGTCACCACCAGGTAGTGGC
		HR1-rv	CCGGCTTAGCCGTA*GGATCC*AATCAACATGGTTCAACGCCCCTC
		HR2-fw	GAACCATGTTGATT*GGATCC*TACGGCTAAGCCGGCTCG
		HR2-rv	tctcgagtctggtcgaccccCTTGCTGCTCCAGTCGTGC
	HiBit tagging	HR1-fw	gagtcgcgagtttaaaccccGCACGCATCGTCTTCGGTG
		HR1-rv	*AACAGCCGCCAGCCGCTCACTCCACTGGATCC*GCCGTAGATCCGCGGCTG
		HR2-fw	*GTGAGCGGCTGGCGGCTGTTCAAGAAGATTAGC*TAAGCCGGCTCGCTCACTTC
		HR2-rv	tctcgagtctggtcgaccccCTTGCTGCTCCAGTCGTGC
aopU	Deletion	HR1-fw	gctaggagtcgcgagtttaaaccccGCTGATGGGTAGCAACCTCG
		HR1-rv	GATCATCATGC*GGATCC*AATGCTTGGCATGTCATAAATCCTTTAAT
		HR2-fw	ATGACATGCCAAGCATT*GGATCC*GCATGATGATCGTTGTCTGGTCA
		HR2-rv	gcatttctcgagtctggtcgaccccAGTAGCTGAGCATGGTCACATC
	HiBit tagging	HR1-fw	gagtcgcgagtttaaaccccGATAGAGATCGCTGCAGCGC
		HR1-rv	*CAGCCGCCAGCCGCTCACTCCACTGGATCC*TGCTTGTTGGATAAGCTTCTCAAAGA
		HR2-fw	*GAGTGAGCGGCTGGCGGCTGTTCAAGAAGATTAGC*TGATGATCGTTGTCTGGTCACTAC
		HR2-rv	gcatttctcgagtctggtcgaccccAGTAGCTGAGCATGGTCACATC
