# Supplementary material for: Cytotoxicity induced by Aeromonas schubertii is orchestrated by a unique set of type III secretion system effectors
Source: Vet Res. 2025 Jun 8;56:113. doi: 10.1186/s13567-025-01548-2 (PMC12147276; doi:10.1186/s13567-025-01548-2)
Supplement: Supplementary file 4 — Additional file 4. Accession numbers of SctN and SctC proteins used for phylogenetic analysis. [file 13567_2025_1548_MOESM4_ESM.pdf]

Additional file 4. Accession numbers of SctN and SctC proteins used for phylogenetic analysis.

**Organism**	**SctN**	**SctC**
Aeromonas salmonicida	ABO92550.1	CAE83117.1
Aeromonas schubertii API1	WP_050666225.1	WP_168929881.1
Aeromonas schubertii API2	WP_050667095.1	WP_019840887.1
Aeromonas veronii API1	MCR4448139.1	ABP51942.1
Aeromonas veronii API2	WP_021229284.1	WP_019840887.1
Bordetella spp.	WP_003820061.1	WP_010930813.1
Edwardsiella ictaluri	ACR68170.1	WP_015870340.1
Escherichia coli E2348/69	WP_000622543.1	AAC38377.1
Escherichia coli O157:H7	WP_000622545.1	WP_000694687.1
Chlamydia pneumoniae	WP_010883345.1	WP_010883340.1
Pseudomonas aeruginosa	WP_003113545.1	WP_003100753.1
Pseudomonas syringae	ABQ88354.1	WP_103689236.1
Rhizobium spp.	ACE93802.1	WP_065114606.1
Salmonella enterica Typhimurium SPI1	WP_000856766.1	WP_000848113.1
Salmonella enterica Typhimurium SPI2	WP_000787213.1	WP_000261284.1
Shigella flexneri	WP_000122616.1	WP_010921673.1
Vibrio parahaemolyticus T3SS-1	WP_005463220.1	WP_011105906.1
Xanthomonas campestris	WP_016851244.1	WP_016851246.1
Yersinia enterocolitica DMO110	AAB69192.1	AGM14903.1
Yersinia enterocolitica DSM 13030	WP_010891217.1	CBY78163.1
