# Supplementary material for: Cytotoxicity induced by Aeromonas schubertii is orchestrated by a unique set of type III secretion system effectors
Source: Vet Res. 2025 Jun 8;56:113. doi: 10.1186/s13567-025-01548-2 (PMC12147276; doi:10.1186/s13567-025-01548-2)
Supplement: Supplementary file 5 — Additional file 5. Phylogenetic analysis of core proteins in API1 and API2 injectisomes. To analyze the phylogenetic relationships of SctN and SctC proteins from Aeromonas API1 and API2, minimum evolution trees based on p-distance were constructed using MEGA11 software. Protein sequences were compared to their orthologs in different bacterial species. The results indicate that AscN and AscC of API1 map within the Ysc family, named after Yersinia spp., while SctN and SctC of API2 belong to the SsaEsc family, which is characteristic for the SPI2-encoded T3SS injectisome in Salmonella enterica serovar Typhimurium. [file 13567_2025_1548_MOESM5_ESM.pdf]

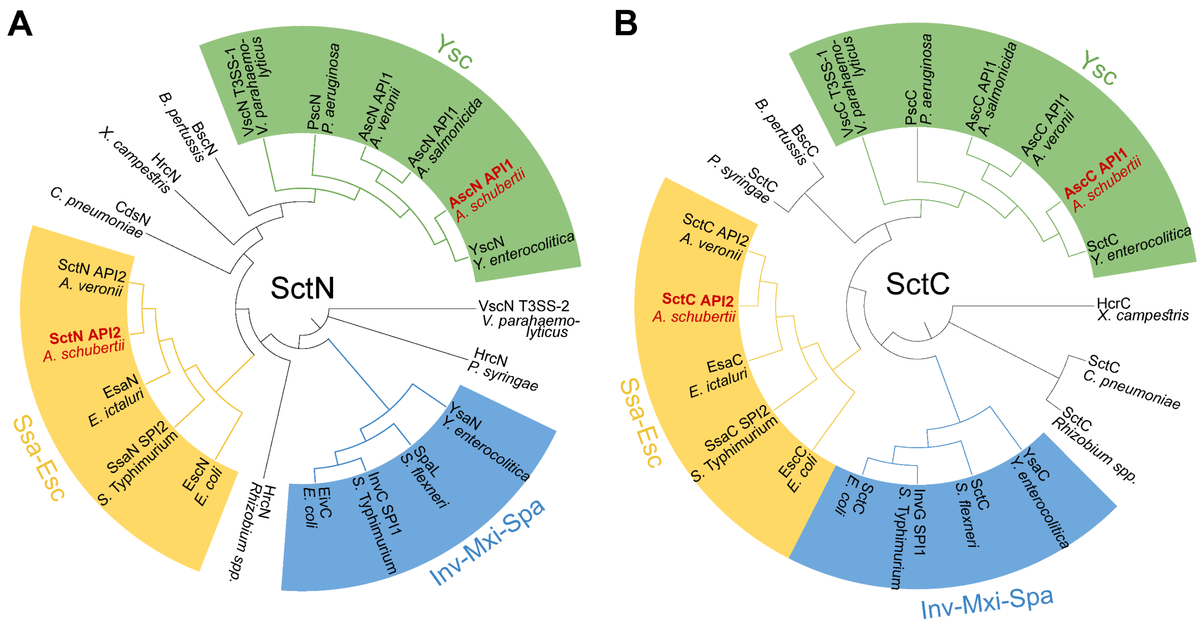

Additional file 5. Phylogenetic analysis of core proteins in API1 and API2 injectisomes.

To analyze the phylogenetic relationships of SctN and SctC proteins from *Aeromonas* API1 and API2, minimum evolution trees based on *p*-distance were constructed using MEGA11 software. Protein sequences were compared to their orthologs in different bacterial species. The results indicate that AscN and AscC of API1 map within the Ysc family, named after *Yersinia* spp., while SctN and SctC of API2 belong to the Ssa-Esc family, which is characteristic for the SPI2-encoded T3SS injectisome in *Salmonella enterica* serovar Typhimurium.
