# Supplementary material for: Cytotoxicity induced by Aeromonas schubertii is orchestrated by a unique set of type III secretion system effectors
Source: Vet Res. 2025 Jun 8;56:113. doi: 10.1186/s13567-025-01548-2 (PMC12147276; doi:10.1186/s13567-025-01548-2)
Supplement: Supplementary file 7 — Additional file 7. Western blot analysis of caspase-3 activation. HeLa cells were either left uninfected or infected with A. schubertii wild-type (WT) or mutant strains lacking the API1 (ΔAPI1) or API2 (ΔAPI2) injectisomes, at an MOI of 10:1. One hour post-infection, the extracellular bacteria were eliminated by the addition of gentamicin. Whole-cell lysates were prepared at indicated time points, separated by SDS-PAGE, and analyzed by immunoblotting using an antibody that detects both the full-length inactive form of caspase-3 (pro-casp 3, 35 kDa) and the cleaved caspase-3 fragment (cleaved casp 3, 17 kDa). βactin (40 kDa) was used as a loading control. HeLa cells treated with 1 µM staurosporine (STS) served as a positive control. Data are representative of 2 independent experiments. [file 13567_2025_1548_MOESM7_ESM.pdf]

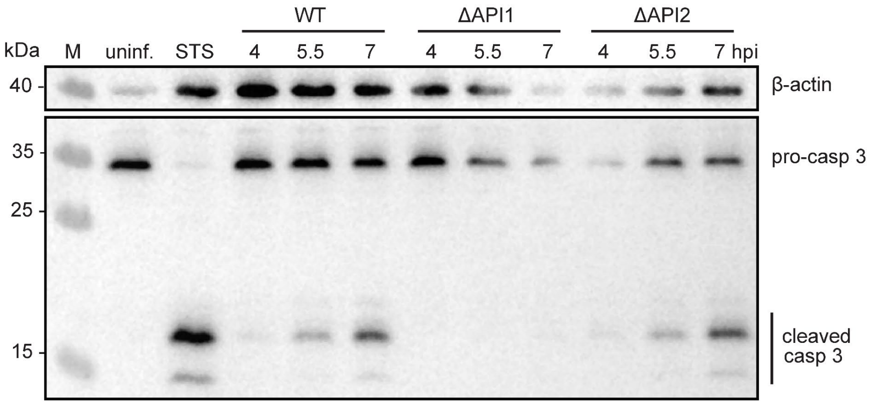

Additional file 7. Western blot analysis of caspase-3 activation.

HeLa cells were either left uninfected or infected with *A. schubertii* wild-type (WT) or mutant strains lacking the API1 (ΔAPI1) or API2 (ΔAPI2) injectisomes, at an MOI of 10:1. One hour post-infection, the extracellular bacteria were eliminated by the addition of gentamicin. Whole-cell lysates were prepared at indicated time points, separated by SDS-PAGE, and analyzed by immunoblotting using an antibody that detects both the full-length inactive form of caspase-3 (pro-casp 3, 35 kDa) and the cleaved caspase-3 fragment (cleaved casp 3, 17 kDa). β-actin (40 kDa) was used as a loading control. HeLa cells treated with 1 µM staurosporine (STS) served as a positive control. Data are representative of 2 independent experiments.
