# Supplementary material for: Cytotoxicity induced by Aeromonas schubertii is orchestrated by a unique set of type III secretion system effectors
Source: Vet Res. 2025 Jun 8;56:113. doi: 10.1186/s13567-025-01548-2 (PMC12147276; doi:10.1186/s13567-025-01548-2)
Supplement: Supplementary file 8 — Additional file 8. Significant changes in the secretome of WT strain compared to the ΔAPI1 derivative in TSB supplemented with 0.5 mM EGTA and 20 mM MgCl2.This table contains Log2-transformed LFQ proteomic data. Significant changes were defined as |fold change| ≥ 4 and -log10P value ≥ 2, corresponding to P ≤ 0.01. [file 13567_2025_1548_MOESM8_ESM.pdf]

**Additional file 8. Significant changes in the secretome of WT strain compared to the ΔAPI1 derivative in TSB supplemented with 0.5 mM EGTA and 20 mM $MgCl_2$.** This table contains $Log_2$-transformed LFQ proteomic data. Significant changes were defined as |fold change| ≥ 4 and $-log_{10}P$ value ≥ 2, corresponding to P ≤ 0.01.

| ID | name | WT | | | ΔAPI1 | | | fold change | $-log_{10}P$ |
|---|---|---|---|---|---|---|---|---|---|
| | | 1 | 2 | 3 | 21 | 22 | 23 | | |
| A0A0W7U3H0 | AopH | 29.487 | 30.665 | 30.148 | 20.322 | 20.004 | 23.046 | 8.976 | 3.030 |
| A0A0W7TWX8 | AopU | 28.131 | 29.879 | 28.489 | 20.664 | 21.342 | 20.558 | 7.978 | 3.771 |
| A0A0W7U479 | AopB | 28.350 | 29.239 | 28.402 | 21.282 | 21.242 | 19.679 | 7.930 | 3.719 |
| A0A0W7TXZ9 | AopJ | 29.581 | 30.632 | 29.951 | 19.867 | 21.902 | 25.241 | 7.718 | 2.074 |
| A0A0W7U449 | AopD | 26.862 | 28.285 | 27.105 | 21.415 | 21.999 | 19.458 | 6.460 | 2.727 |
| A0A0W7U4M4 | AopO | 26.043 | 27.006 | 26.415 | 20.565 | 21.121 | 18.767 | 6.337 | 2.939 |
| A0A0W7U448 | AcrV | 28.809 | 30.155 | 29.583 | 22.474 | 24.222 | 24.128 | 5.908 | 2.994 |
| A0A0W7U426 | AopN | 29.102 | 30.153 | 28.794 | 23.614 | 22.387 | 25.096 | 5.651 | 2.511 |
| A0A0W7U434 | AscF | 25.967 | 27.214 | 26.097 | 21.116 | 22.927 | 19.793 | 5.147 | 2.185 |
| A0A0W7U489 | AopR | 24.777 | 25.851 | 24.623 | 20.050 | 22.250 | 19.188 | 4.588 | 2.009 |
| A0A0W7TWU2 | AopI | 25.030 | 26.012 | 25.703 | 20.761 | 21.017 | 21.270 | 4.565 | 3.827 |
| A0A0W7U4B3 | AscP | 24.945 | 26.067 | 25.435 | 19.468 | 22.031 | 21.774 | 4.392 | 2.127 |
| A0A0W7U4D9 | AopT | 26.839 | 28.126 | 27.233 | 22.270 | 23.830 | 23.568 | 4.177 | 2.612 |
| A0A0W7U2M6* | AopL | 27.453 | 28.156 | 27.524 | 22.982 | 25.472 | 24.024 | 3.552 | 2.031 |

* A potential effector homologous to the VopQ effector of *Vibrio parahaemolyticus* does not meet the specified fold
