# Supplementary material for: Cytotoxicity induced by Aeromonas schubertii is orchestrated by a unique set of type III secretion system effectors
Source: Vet Res. 2025 Jun 8;56:113. doi: 10.1186/s13567-025-01548-2 (PMC12147276; doi:10.1186/s13567-025-01548-2)
Supplement: Supplementary file 9 — Additional file 9. Sequence alignment of AopI, AopJ, and AopL with homologous T3SS effectors. ExoY from Pseudomonas aeruginosa (WP_003115517.1) in (A), OspF from Shigella flexneri (HCR8314926.1) in (B), and VopQ from Vibrio parahaemolyticus (WP_005464333.1) in (C) were used for comparison. Amino acid sequences were aligned using the Clustal Omega online alignment tool available at the Uniprot website and visualized with the percentage identity scheme in Jalview. [file 13567_2025_1548_MOESM9_ESM.pdf]

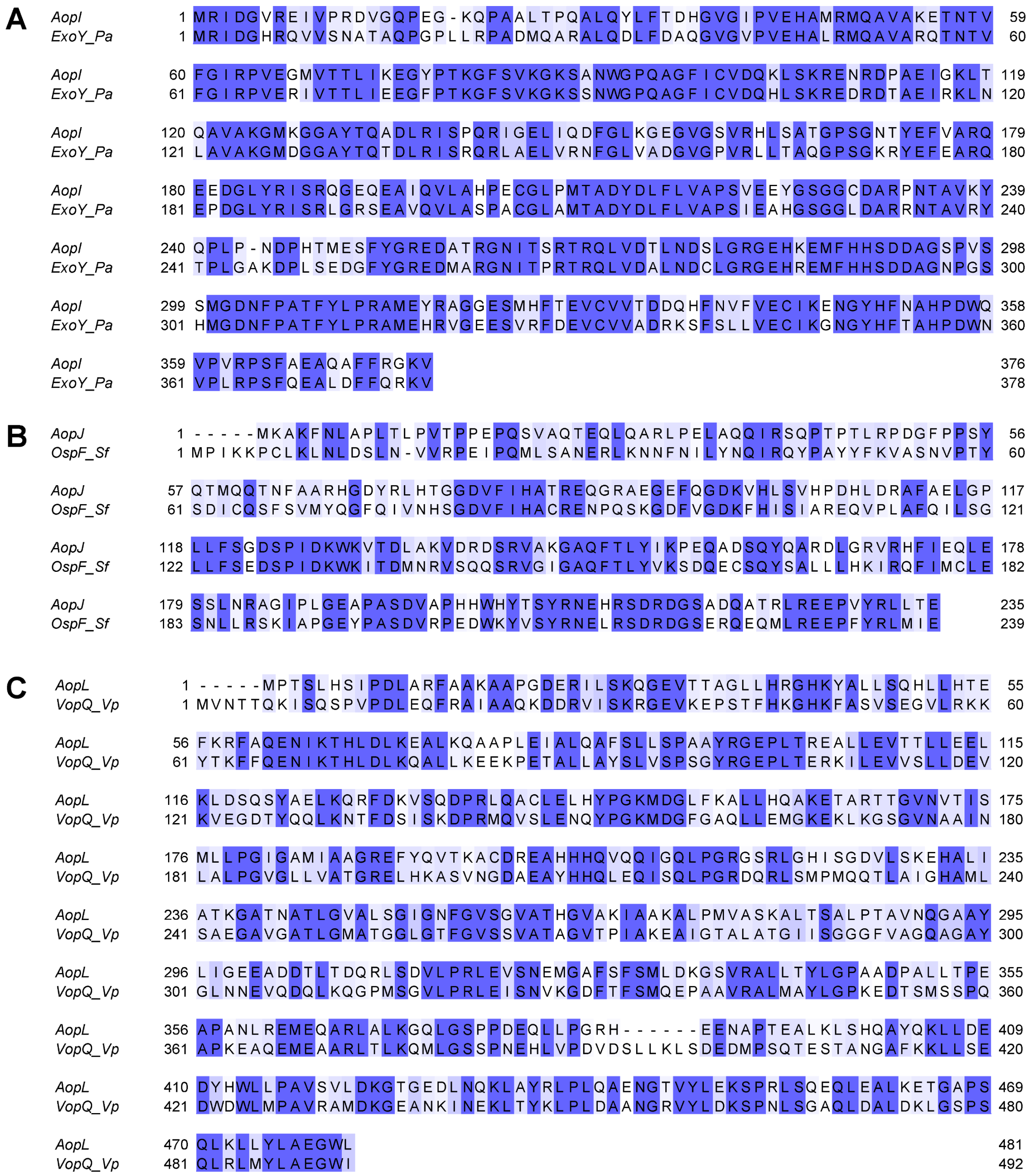

Additional file 9. Sequence alignment of AopI, AopJ, and AopL with homologous T3SS effectors.

ExoY from *Pseudomonas aeruginosa* (WP_003115517.1) in (A), OspF from *Shigella flexneri* (HCR8314926.1) in (B), and VopQ from *Vibrio parahaemolyticus* (WP_005464333.1) in (C) were used for comparison. Amino acid sequences were aligned using the Clustal Omega online alignment tool available at the Uniprot website (https://www.uniprot.org/align) and visualized with the percentage identity scheme in Jalview.
