# Supplementary material for: Cytotoxicity induced by Aeromonas schubertii is orchestrated by a unique set of type III secretion system effectors
Source: Vet Res. 2025 Jun 8;56:113. doi: 10.1186/s13567-025-01548-2 (PMC12147276; doi:10.1186/s13567-025-01548-2)
Supplement: Supplementary file 10 — Additional file 10. Candidate effectors of the API1 injectisome. Amino acid sequences are provided. [file 13567_2025_1548_MOESM10_ESM.pdf]

Additional file 10. Candidate effectors of the API1 injectisome. Amino acid sequences are provided.

AopH (homologous to *Yersinia* YopH and *Salmonella* SptP protein tyrosine phosphatase);<br>A0A0W7U3H0_9GAMM protein-tyrosine-phosphatase OS=*Aeromonas schubertii* OX=652<br>GN=ATO46_16020 PE=4 SV=1
MALSLQALHSKVTQLAQNNGELTGKFKNDSLAAHKEGSFQGLTVSSGARPTERAFAQEVLKHLQHV<br>SLNDGEVRGLTEGSRYAQSNFELHYDGKGVQLRGLHSDQLTLKDAKILLDAALRQQGREEAPPLPP<br>RSAPVRTDRPALAHQHERLMGTAPRLVAAPPADPCGSAQKQQLSDRLKALQDQLSPGNDPDRYQQ<br>GSGVNRFRDIQANKATAVREDLNANYVQVGAHRSIACQYPLQAQLESHMQMLFDNRTPVLAVLASA<br>SEIDTPGNKMPDYFRQDGQYGQMKVKSALHHSVDLGRGIQADVYHMTLSQPNSGKKGIVVPVVHVS<br>NWPDKQAVGTDVSDNLARLLEQTTQEKKDMYTRAGSSAVGDDNKLLPVIHCRAGVGRTGQVIGTM<br>AMNDPRNGALSVEDVVGEMRQHRNGIMVQTQGQLDELVNLAQQQGRSLLRA
AopO (homologous to *Yersinia* YopO, also known as YpkA, serine/threonine protein kinase);<br>A0A0W7U4M4_9GAMM non-specific serine/threonine protein kinase OS=*Aeromonas schubertii*<br>OX=652 GN=ATO46_13315 PE=4 SV=1
MKIIGTATPSITLSQAHERVANHGQHPVGELNIEGKRYRIVDNQVLRLNPHGGIARFREGVGKLFSGQ<br>APDTSYARALTETLHAARKAAPKSPGGEAPQGIGGLFGLKPQTRLPLGWKGEPLPGAPSLEGMRVA<br>ETDKFAEGESHISIVETRDKQRLVAKIERSVAEGHLQGELEAYQHIYQSAGKHPNLGNVHGMAVVPY<br>GSRKEEALLMDEVDGWRGSDTMRTLTDNWKQGKVSSEEYWGTVKFIAHRLLDVTGHLAKAGIVHN<br>DIKPGNVVFDKHSGEPVVIDLGLHSRPGEQPKGFTDSFKAPELAVGSPSASEKSDVFLVVSTLLHGIE<br>GFERDLERKPNQGLSLTRGAAHGLDERGNPVHRPGVAGVETAYTRFVDQIIGAPAELRPDSAEARL<br>HEFLSDGAIDEGRARQILKETLSGELAASPADARRVTPRKIRELSDTLRLHLSSASTRQLNVGMALSD<br>LAAMSAVLDKAERQEFADQGQLKSFNSLILKGYGVIARYVKGELGESKTPQGEPSPQLRGNIMKSVA<br>EPTLKQIQGQLAQRHGLVDIATLERSHHHLETLLTVLVPSSPQEKVSPEAYDFLNRVAEVKGSLGARL<br>DDLKGQQQRAHGELSTLMRAATAWAGDARQALQRFDSIRPVVKFGSDQDTAVHRSMIAAHAATTLQ<br>EVAGFAGEMRHFAAAATPLLTQLGRSTLADEGLTFQREQLRELATVAERLTRLSQEWIR
AopI (homologous to *Pseudomonas* ExoY nucleotidyl cyclase); A0A0W7TWU2_9GAMM Adenylate<br>cyclase OS=*Aeromonas schubertii* OX=652 GN=ATO46_11390 PE=4 SV=1
MRIDGVREIVPRDVGQPEGKQPAALTPQALQYLFTDHGVGIPVEHAMRMQAVAKETNTVFGIRPVEG<br>MVTTLIKEGYPTKGFSVKGKSANWGPQAGFICVDQKLSKRENRDPAEIGKLTQAVAKGMKGGAYTQ<br>ADLRISPQRIGELIQDFGLKGEGVGSVRHLSATGPSGNTYEFVARQEEDGLYRISRQGEQEAIQVLAH<br>PECGLPMTADYDLFLVAPSVEEYGSGGCDARPNTAVKYQPLPNDPHTMESFYGREDATRGNITSRT<br>RQLVDTLNDSLGRGEHKEMFHHSDDAGSPVSSMGDNFPATFYLPRAMEYRAGGESMHFTEVCVVT<br>DDQHFNVFVECIKENGYHFNAHPDWQVPVRPSFAEAQAFFRGKV
AopJ (homologous to *Shigella* OspF phosthreoninelyase); A0A0W7TXZ9_9GAMM OS=*Aeromonas*<br>*schubertii* OX=652 GN=ATO46_09880 PE=3 SV=1
MKAKFNLAPLTLPVTPPEPQSVAQTEQLQARLPELAQQIRSQPTPTLRPDGFPPSYQTMQQTNFAAR<br>HGDYRLHTGGDVFIHATREQGRAEGEFQGDKVHLSVHPDHLDRAFAELGPLLFSGDSPIDKWKVTD<br>LAKVDRDSRVAKGAQFTLYIKPEQADSQYQARDLGRVRHFIEQLESSLNRAGIPLGEAPASDVAPHH<br>WHYTSYRNEHRSDRDGSADQATRLREEPVYRLLTE
AopL (homologous to *Vibrio* VopQ); A0A0W7U2M6_9GAMM Cation transporter OS=*Aeromonas*<br>*schubertii* OX=652 GN=ATO46_04060 PE=4 SV=1
MPTSLHSIPDLARFAAKAAPGDERILSKQGEVTTAGLLHRGHKYALLSQHLLHTEFKRFAQENIKTHL<br>DLKEALKQAAPLEIALQAFSLLSPAAYRGEPLTREALLEVTTLLEELKLDSQSYAELKQRFDKVSQDPR<br>LQACLELHYPGKMDGLFKALLHQAKETARTTGVNVTISMLLPGIGAMIAAGREFYQVTKACDREAHH<br>HQVQQIGQLPGRGSRLGHISGDVLSKEHALIATKGATNATLGVALSGIGNFGVSGVATHGVAKIAAKA<br>LPMVASKALTSALPTAVNQGAAYLIGEEADDTLTDQRLSDVLPRLEVSNEMGAFSFSMLDKGSVRAL<br>LTYLGPAADPALLTPEAPANLREMEQARLALKGQLGSPPDEQLLPGRHEENAPTEALKLSHQAYQKL<br>LDEDYHWLLPAVSVLDKGTGEDLNQKLAYRLPLQAENGTVYLEKSPRLSQEQLEALKETGAPSQLKL<br>LYLAEGWL
AopT; A0A0W7U4D9_9GAMM Lipase_3 domain-containing protein OS=*Aeromonas schubertii*<br>OX=652 GN=ATO46_13805 PE=4 SV=1
MLINSALGHVAIQNRPATSEAPASASGLRQTSSVAAPLANPRAPLDTPAAASGPDLRQIKATKVSLDD<br>KPAIDAIKTTLIADAALANFPYTQDMGELGGATAKGWGTGSGHLLQALSVGPGQKNIGDNGILTKSGL<br>TAYLFTNESTREARIVFGGTTSGEKAGDLNTRTLGNLATTAKQWLANIKNVFSRTPDSYKEAAKLVSD<br>VKDHLPAGYALSVSGHSKGGGESAYAAMMLGAKTGEPVKSINFSSAELGAKLKQNIASELAKGGIQP<br>DAIAGKFEELGKEILHIKIKGDPVPNMHKFFGSISHIGRTLTLPNDNKSVAHLSEHVDFFSRVASWSHA<br>GSMSHHQLINQPRIYG

AopU; A0A0W7TWX8_9GAMM Rho-GAP domain-containing protein OS=*Aeromonas schubertii* OX=652 GN=ATO46_11430 PE=4 SV=1
MPSIASQVQILPPQVHQELTEKLKGEKSPAGVANVELGNQMHTISVVETSCDAKHVGEGRKSFLSSV<br>KDFFNSVRDAFINFFSKASEPTKGSIEIAAAPVMDKHAAVVAQNKEIMGIFLQNHDFLKTEGIMRISAAK<br>TELDLLSAGKKDLQDATGVELAALFKKNIREHCSPADMKAFEQTFLDYQNNNQLPLVSDLPEMAQDA<br>IMLAKEVAKYQGENHMTASNLAIVMAPNLMSTELLMQGKTLEFNTFFEKLIQQA
