# Supplementary material for: Cytotoxicity induced by Aeromonas schubertii is orchestrated by a unique set of type III secretion system effectors
Source: Vet Res. 2025 Jun 8;56:113. doi: 10.1186/s13567-025-01548-2 (PMC12147276; doi:10.1186/s13567-025-01548-2)
Supplement: Supplementary file 11 — Additional file 11. Response of candidate effector proteins to low Ca2+/ high Mg2+ concentrations. Cultures of WT reporter strains (WTHiBiT) and mutant derivatives lacking API1 (ΔAPI1HiBiT) or API2 (ΔAPI2HiBiT) injectisomes were grown in TSB medium without and with supplementation of 0.5 mM EGTA and 20 mM MgCl2. The amount of candidate effectorHiBiT in each fraction was expressed as a percentage of the total candidate effectorHiBiT present in the culture. Data are representative of 3 independent experiments. ND, not determined. [file 13567_2025_1548_MOESM11_ESM.pdf]

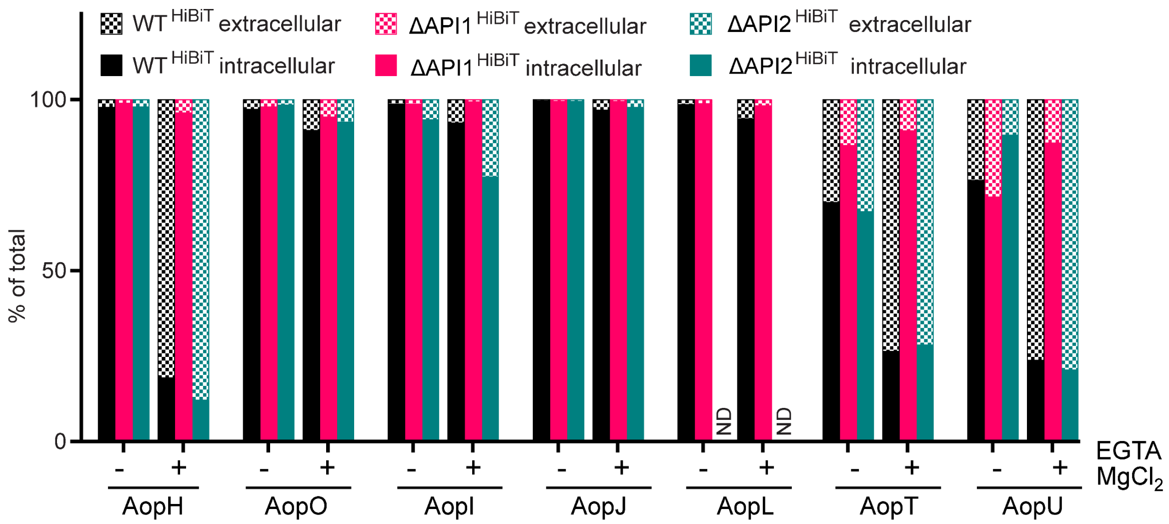

Additional file 11. Response of candidate effector proteins to low Ca^{2+}/ high Mg^{2+} concentrations.

Cultures of WT reporter strains (WT^{HiBiT}) and mutant derivatives lacking API1 ($\Delta API1^{HiBiT}$) or API2 ($\Delta API2^{HiBiT}$) injectisomes were grown in TSB medium without and with supplementation of 0.5 mM EGTA and 20 mM $MgCl_2$. The amount of candidate $effector^{HiBiT}$ in each fraction was expressed as a percentage of the total candidate $effector^{HiBiT}$ present in the culture. Data are representative of 3 independent experiments. ND, not determined.
