# Supplementary material for: Cytotoxicity induced by Aeromonas schubertii is orchestrated by a unique set of type III secretion system effectors
Source: Vet Res. 2025 Jun 8;56:113. doi: 10.1186/s13567-025-01548-2 (PMC12147276; doi:10.1186/s13567-025-01548-2)
Supplement: Supplementary file 12 — Additional file 12. Distribution and similarity of the identified T3SS effectors across genomes of A. schubertii strains. The heatmap displays the presence and percentage similarity of T3SS effectors identified in A. schubertii ATCC 43700 across other available A. schubertii genomes. Absence of an effector is indicated by black / ND (not detected). The following genomes were analyzed: the type strain A. schubertii ATCC 43700 (NCBI RefSeq: GCF_001481395.1), originally isolated from a forehead abscess in Texas, United States. Alternative designations for this strain include CECT4240, CDC 2446-81, CCTM La 3016, CCUG 27820, DSM 4882, JCM 7373, LMG 9074, and NCIMB 13161. A different passage of this strain is also deposited in NCBI under its alternative name CECT4240 (NCBI RefSeq GCF_000820105.1). Strain A40 (NCBI RefSeq: GCF_045983075.1) was isolated from a case of Aeromonas septicemia in Asian sea bass (Lates calcarifer) in Thailand. Strains CHULA2021a (NCBI RefSeq: GCF_020089825.1) and CHULA2021b (NCBI RefSeq: GCF_020089835.1) were recovered from mass mortality events in Asian sea bass (Lates calcarifer) in Thailand. Additionally, strain LF1708 (NCBI RefSeq: GCF_004919485.1) was isolated from diseased Nile tilapia (Oreochromis niloticus), whereas strain WL1483 (NCBI RefSeq: GCF_001447335.1) was isolated from diseased snakehead fish (Channa argus), both in China. [file 13567_2025_1548_MOESM12_ESM.pdf]

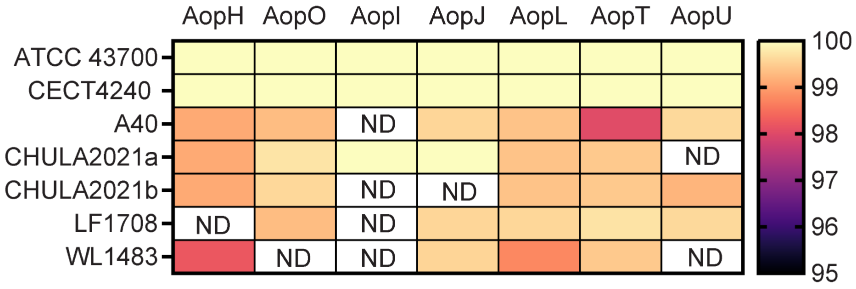


**Additional file 12. Distribution and similarity of the identified T3SS effectors across genomes of *A. schubertii* strains.**

The heatmap displays the presence and percentage similarity of T3SS effectors identified in *A. schubertii* ATCC 43700 across other available *A. schubertii* genomes. Absence of an effector is indicated by black / ND (not detected). The following genomes were analyzed: the type strain *A. schubertii* ATCC 43700 (NCBI RefSeq: GCF_001481395.1), originally isolated from a forehead abscess in Texas, United States. Alternative designations for this strain include CECT4240, CDC 2446-81, CCTM La 3016, CCUG 27820, DSM 4882, JCM 7373, LMG 9074, and NCIMB 13161. A different passage of this strain is also deposited in NCBI under its alternative name CECT4240 (NCBI RefSeq GCF_000820105.1). Strain A40 (NCBI RefSeq: GCF_045983075.1) was isolated from a case of *Aeromonas* septicemia in Asian sea bass (*Lates calcarifer*) in Thailand. Strains CHULA2021a (NCBI RefSeq: GCF_020089825.1) and CHULA2021b (NCBI RefSeq: GCF_020089835.1) were recovered from mass mortality events in Asian sea bass (*Lates calcarifer*) in Thailand. Additionally, strain LF1708 (NCBI RefSeq: GCF_004919485.1) was isolated from diseased Nile tilapia (*Oreochromis niloticus)*, whereas strain WL1483 (NCBI RefSeq: GCF_001447335.1) was isolated from diseased snakehead fish (*Channa argus*), both in China.
